# Machine learning water quality inversion via optimal band selection from highly correlated subsets

**DOI:** 10.1016/j.isci.2026.117027

**Published:** 2026-08-03

**Authors:** Xiaonan Yang, Jiansheng Wang, Li Sun, Songjia Zhang, Kun Ding, Zhiping He, Qingli Li

**Affiliations:** 1 Shanghai Key Laboratory of Multidimensional Information Processing, East China Normal University, Shanghai 200241, China; 2 College of Environmental Science and Engineering, Tongji University, Shanghai 200092, China; 3 Shanghai Institute of Technical Physics of the Chinese Academy of Sciences, Shanghai 200083, China

**Keywords:** water quality inversion, band selection, UAV multispectral remote sensing, spatial heterogeneity

## Abstract

Rapid urbanization has triggered severe water contamination in urban fluvial ecosystems, making efficient water quality monitoring and precise retrieval an urgent research priority. Taking Shanghai’s Lianqi River as the study area, this paper targets COD and ammonia nitrogen with distinct concentration ranges. On the basis of UAV multispectral remote sensing observations, this work explores water quality retrieval via diverse feature screening strategies and machine learning approaches. Exhaustive search is adopted to generate abundant feature combinations, and six algorithms including CatBoost, XGBoost, LightGBM, KNN, RF, and MBLERM are employed to build massive optimized model clusters. A multi-dimensional feature evaluation system is constructed to assess feature properties from correlation, distance, and spectral morphology perspectives. Results show that linear correlation cannot determine model precision; model accuracy declines beyond seven features due to dimensional catastrophe; pollutant spatial distribution presents obvious heterogeneity closely associated with land-use patterns.

## Highlights

• Exhaustive feature search and six ML algorithms built 5,802 evaluation models
• Multi-model analysis reveals synergistic adaptation between features and algorithms
• Ensemble models show superior robustness against high-dimensional redundancy

## Graphical abstract

**Figure:**
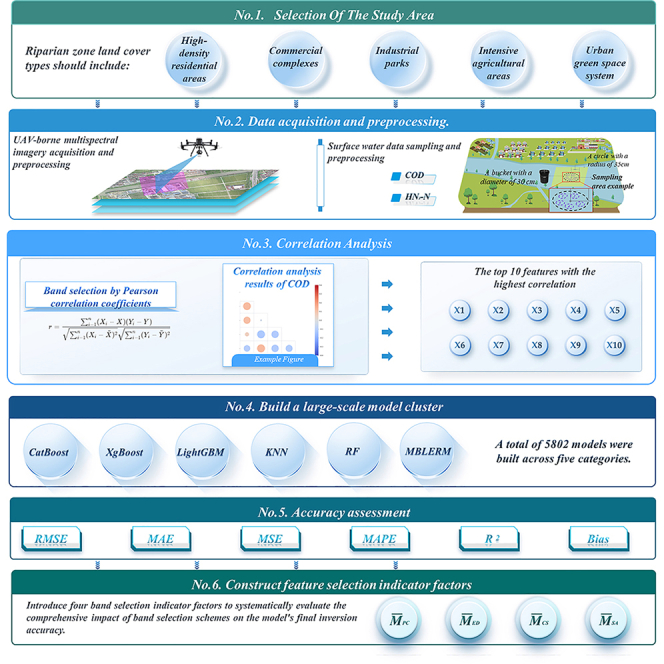

## Teaser

Environmental monitoring; Remote sensing; Machine learning

## Introduction

Urban rivers are an integral component of urban ecosystems, offering irreplaceable ecological functions and cultural values.^1^^,^^2^ As the core carrier of urban ecological corridors, urban rivers not only sustain regional biodiversity and regulate local climate but also serve as crucial freshwater resources for sustainable urban development.^3^^,^^4^^,^^5^ Simultaneously, rivers carry the historical legacy of cities and provide important spaces for leisure and recreation for citizens. However, against the backdrop of rapid urbanization, urban river ecosystems face severe challenges. The increasing surface imperviousness, continuous expansion of urban construction land, and intensifying human activities have collectively led to significant fluctuations in the water quality of urban rivers.^6^^,^^7^ Therefore, achieving rapid and accurate monitoring of urban river water quality has become an urgent task for water environment management and conservation.^8^

Traditional water quality monitoring primarily relies on ground sampling analysis and automatic monitoring stations. While this method offers high accuracy, it is inherently limited by high costs, inefficiency, and the inability to capture spatially continuous variations in water quality.^9^ The rise of drone remote sensing technology has provided a technical pathway to overcome these limitations. With its high spatial and temporal resolution, as well as flexibility and mobility, drone technology is particularly well-suited for detailed monitoring of medium-to small-scale water bodies in urban environments.^10^^,^^11^^,^^12^ A key challenge in constructing high-precision water quality inversion models lies in effectively selecting the spectral bands or band combinations most sensitive to specific water quality parameters from the multispectral data acquired by drones.^13^

For a long time, the field of water quality remote sensing has generally followed the “high correlation band priority” selection strategy, which involves analyzing the Pearson correlation coefficient between spectral bands and water quality parameters.^14^ Spectral bands with higher correlation coefficients are considered to have stronger predictive capabilities.^15^^,^^16^^,^^17^^,^^18^ This Pearson correlation-based band selection paradigm may be effective in traditional water quality inversion models based on statistical methods such as linear regression. However, in the current context dominated by complex machine learning algorithms, such as ensemble learning,^19^^,^^20^^,^^21^^,^^22^^,^^23^ it remains a scientific question whether this method can fully meet the demands for high-precision and robust modeling. Traditional methods assume a stable linear relationship between spectral bands and water quality parameters, whereas the core advantage of machine learning models (e.g., XGBoost^22^^,^^23^^,^^24^^,^^25^^,^^26^ and RF^27^^,^^28^^,^^29^) lies in their ability to autonomously uncover complex nonlinear interactions and higher-order effects between band features and target parameters.^30^^,^^31^ Relying solely on linear correlation coefficients as a selection criterion may not only overlook nonlinear feature combinations that are crucial to model performance but could also introduce redundant bands, leading to the “curse of dimensionality,” which ultimately limits the improvement of model generalization ability. In other words, the spectral bands most highly correlated with water quality parameters may not necessarily yield the most optimal predictive model.^32^

In remote sensing-based water quality estimation, feature selection typically relies on a two-stage framework, where a subset of spectral bands is first selected using model-agnostic criteria—such as Pearson correlation coefficient, recursive feature elimination (RFE), or sequential forward selection (SFS)—before training a single machine learning model.^33^ While this decoupled approach simplifies model training, integrating band selection and regression into a unified optimization framework remains a persistent challenge in the field.^34^ Although computationally efficient, this approach presents a critical limitation: it decouples feature selection from the model learning process, neglecting the fundamental differences in how diverse machine learning algorithms respond to feature dimensionality, redundancy, and interactive structures.^35^ For instance, K-nearest neighbors (KNN) is highly susceptible to the “curse of dimensionality” and prone to overfitting in high-dimensional spaces, whereas tree-based ensemble models, such as random forest (RF) and XGBoost, exhibit inherent robustness to irrelevant or redundant features. Consequently, a band combination identified through a specific, fixed feature selection framework often demonstrates inconsistent or significantly fluctuating performance when transferred across different regression models.^36^ This issue stems from the intrinsic mechanisms of heuristic search strategies (e.g., SFS or RFE).^37^ Prioritizing computational efficiency, these methods explore only constrained sub-regions of the high-dimensional feature space, failing to comprehensively capture the optimal feature structures tailored to a specific model.^38^ This limitation becomes particularly pronounced in remote sensing water quality inversion. The optical properties of water bodies are typically governed by complex, nonlinear synergistic effects among multiple biochemical constituents. If feature selection is divorced from the learning mechanism of the chosen model, it struggles to effectively represent these highly coupled physical processes, thereby constraining the model’s generalizability and transferability.^39^^,^^40^^,^^41^

To address these shortcomings, this study proposes a joint optimization framework that abandons the traditional pre-selection step and instead, systematically evaluates all possible combinations of 3–9 bands from the 10 available spectral bands (a total of 967 combinations). For each combination, we train and validate six different machine learning algorithms, resulting in 5,802 models for each water quality parameter. Therefore, this study shifts its perspective to focus on the joint evolutionary dynamics between feature dimensions and model architectures. Rather than seeking static, model-agnostic band combinations, we investigate the dynamic synergies formed between specific feature subsets and diverse machine learning architectures across complex environmental gradients.^42^^,^^43^ This design enables us to quantitatively dissect the intrinsic trade-off between computational complexity and modeling capacity.

Selecting specific water quality parameters for remote sensing monitoring is critical, as they directly reflect the ecological health and pollution stress levels of water bodies. This study focuses on two key indicators: chemical oxygen demand (COD) and ammonia nitrogen (NH_3_-N), which are widely recognized as core metrics for assessing pollution in urban rivers and play indispensable roles in environmental management and ecological risk early warning.^44^^,^^45^^,^^46^ (1) COD represents the total load of organic pollutants in water and serves as an important measure of organic pollution. High COD concentrations typically indicate substantial inputs of domestic sewage, industrial effluent, or surface runoff carrying organic matter.^9^ Such inputs not only increase biochemical oxygen demand and accelerate dissolved oxygen depletion but may also cause water discoloration and malodor, severely threatening aquatic life and disrupting ecosystem balance.^47^^,^^48^ In urban rivers, organic pollution often coincides with the enrichment of colored dissolved organic matter (CDOM), which exhibits strong absorption in the blue spectral region (∼475 nm), systematically reducing water reflectance and generating identifiable indirect optical signals in multispectral imagery.^49^ (2) NH_3_-N primarily originates from agricultural runoff, domestic wastewater, and industrial discharge, representing a key form of nitrogen pollution. Elevated NH_3_-N concentrations can easily trigger eutrophication, promoting explosive algal growth (particularly cyanobacteria), leading to harmful algal blooms, diurnal dissolved oxygen fluctuations, and even anoxic conditions. Such processes ultimately deteriorate water quality and may disrupt entire aquatic ecosystems. Therefore, dynamic monitoring of NH_3_-N is essential for identifying pollution sources, assessing eutrophication risk, and informing targeted management strategies.^50^^,^^51^ *In situ*, areas with high NH_3_-N often coincide with low dissolved oxygen and high organic loads, commonly manifesting as darker water (reduced reflectance) or early algal responses (e.g., enhanced green-band reflectance).^52^ While these changes are not intrinsic spectral features of NH_3_-N, they constitute indirect signals detectable by remote sensing.^53^^,^^54^ Although COD and NH_3_-N are not inherently optically active constituents, in urban rivers dominated by domestic pollution—such as the Lianqi River—where water quality-optical relationships are relatively stable, their concentration variations exhibit significant and reproducible indirect coupling with water spectral responses.^55^^,^^56^ By leveraging the fine-scale characterization of micro-scale pollution patches provided by 10 cm ultra-high-resolution unmanned aerial vehicle (UAV) multispectral imagery, combined with the capacity of machine learning algorithms to capture complex nonlinear mapping relationships, this study demonstrates that remote sensing retrieval of COD and NH_3_-N is both scientifically justified and technically feasible.

It is also noteworthy that COD and NH_3_-N exhibit significant differences in concentration levels, spectral response characteristics, and sources of pollution. This heterogeneity makes them ideal candidates for testing the generalization ability of remote sensing inversion models.^51^^,^^57^ To systematically evaluate the applicability of remote sensing technology in complex urban water environments and overcome the limitations of traditional band selection methods based on simple correlation, this study focuses on the Lianqi River Basin in Shanghai as a typical research area, conducting an in-depth investigation on the following three aspects:

(1) This study focuses on two water quality parameters with significant heterogeneity, COD and NH_3_-N. Exhaustive searches were conducted for combinations of 3–9 bands from the available 10 feature bands. For each parameter, a total of 5,802 models were constructed, encompassing six algorithms: CatBoost, XGBoost, LightGBM, KNN, RF, and MBLERM. This approach systematically reveals the synergistic adaptation patterns between band combinations and algorithm performance. Supplementary experiments further demonstrate that the method is also applicable to the retrieval of chlorophyll-a (Chl-a), validating its suitability across different water quality parameters.
(2) A multi-dimensional evaluation system was developed, incorporating the average correlation coefficient ($\bar{M}$_*PC*_), average Euclidean distance ($\bar{M}$_*ED*_), average cosine similarity ($\bar{M}$_*CS*_), and average spectral angle ($\bar{M}$_*SA*_). The analysis indicates that high linear correlation between features and water quality parameters is not a decisive factor for inversion accuracy. It further confirms the advantage of ensemble learning algorithms in capturing nonlinear data features.
(3) Based on the optimal models, spatial inversions reveal distinct spatial patterns in COD and NH_3_-N concentrations in the Lianqi River Basin. High COD areas (>12 mg/L) are concentrated in the southwest agricultural sections, while NH_3_-N pollution hotspots (>3 mg/L) are found in the northeastern industrial and residential areas, showing significant spatial heterogeneity.

## Results

**Figure 1:**
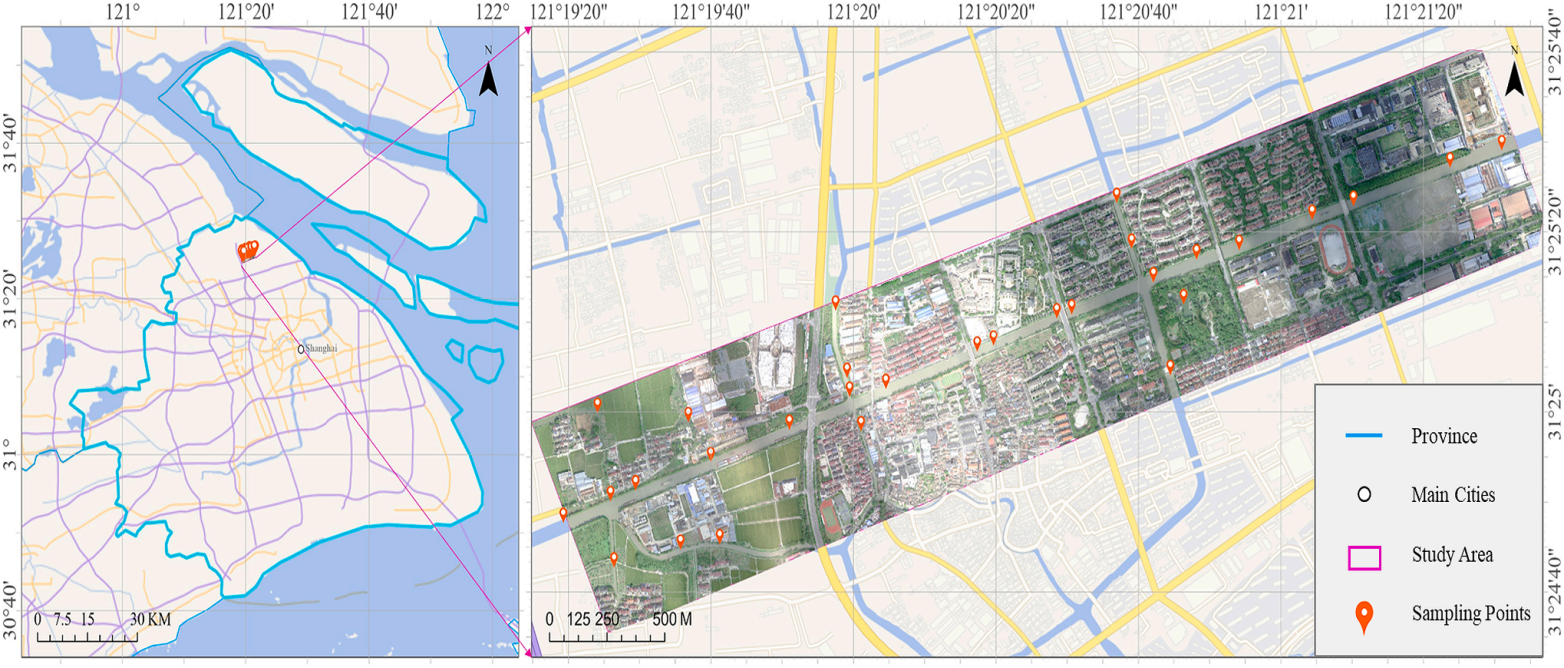
Geographic location schematic diagram of the study area and distribution of sampling sites The Lianqi River in Shanghai (3.7 km long and covering an area of 2.96 km^2^; 121°21′13″E−121°21′38″E, 31°24′34″N–31°25′42″N) flows from Jiading to Baoshan before merging into the Yangtze River; surrounded by diverse land uses, it represents a typical urban-suburban composite ecosystem with complex spatial heterogeneity. Left: shows the regional location of the study area within Shanghai at a map scale of 1:830,000, with the embedded scale bars representing an actual length of 30 km. Right: displays the detailed map of the Lianqi River study area and sampling point distribution at a map scale of 1:12,000, with the embedded scale bars representing an actual length of 500 m.

On the basis of UAV multispectral remote sensing data, this section systematically evaluates the river water quality inversion accuracy of six machine learning algorithms across diverse feature combinations. It quantifies the impacts of feature correlation, spatial distance, and spectral morphology on model prediction stability, thereby identifying the optimal feature combination scheme for regional river water quality monitoring. Furthermore, it reveals the spatially heterogeneous distribution patterns of key water pollutants within the study area and elucidates the responsive relationships between the spatial differentiation of pollutants and land-use patterns. Figure 1 illustrates the geographical scope of the study area alongside the spatial distribution of all field sampling sites.

### Band combinations and water quality parameters selection

For each water quality parameter, a total of 2,880 correlation coefficients were obtained, comprehensively characterizing the statistical relationships between various feature combinations and the water quality parameters. After eliminating statistically insignificant results, weakly correlated features, and those exhibiting numerical instability (e.g., overflow or invalid values) from the correlation analysis, the top 10 feature combinations showing the highest correlations with each water quality parameter were ultimately selected as model inputs. The characteristic features used for modeling different water quality parameters and their corresponding correlation results are presented in Table 1.

**Table 1:** Feature selection and correlation analysis

Water quality parameter	Type	Correlation analysis				
COD	feature operation formula	*X1* = *B5/(B1 - B3)*	*X2* = *B2/(B1 - B3)*	*X3* = *B4/(B1 - B3)*	*X4* = *B1/(B1 - B3)*	*X5* = *B3/(B4 - B1)*
	correlation coefficient	0.33	0.31	0.31	0.30	0.29
	feature operation formula	*X6* = *(B1- B2* + *B3)/(B1* + *B2-B3)*	*X7* = *B2/(B4 - B1)*	*X8* = *B4/(B4 - B1)*	*X9* = *B5/(B4 – B1)*	*X10* = *B3/(2∗B2- B3)*
	correlation coefficient	0.28	0.28	0.28	0.27	0.26
NH3-N	feature operation formula	*X1* = *(B5- B2* + *B3)/(B5* + *B2-B3)*	*X2* = *(B4- B5* + *B2)/(B4* + *B5-B2)*	*X3* = *B3/(B3 - B1)*	*X4* = *B2/(B3 - B1)*	*X5* = *B2/(B4 - B3)*
	correlation coefficient	0.69	0.66	0.42	0.41	0.40
	feature operation formula	*X6* = *B1/(B4 - B3)*	*X7* = *B4/(B4 - B3)*	*X8* = *B5/(B4 - B3)*	*X9* = *ln(B4)*	*X10* = *ln(B3)* + *ln(B4)*
	correlation coefficient	0.39	0.39	0.38	0.38	0.37

### Impact of feature combinations on the accuracy of COD retrieval in aquatic environments

This study systematically assessed the impact of spectral feature combinations on COD inversion using six machine learning models: CatBoost, XGBoost, LightGBM, KNN, RF, and MBLERM (Table 2). A total of 5,802 models were developed. CatBoost consistently achieved the highest accuracy with 5–7 features (root-mean-square error [RMSE] = 0.42–0.44, coefficient of determination [R^2^] = 0.93), with features *X*3, *X*6, and *X*7 appearing most frequently in the optimal combinations. Model performance improved with increasing feature numbers up to seven but declined beyond this threshold, indicating the detrimental effect of redundant features. Traditional models such as KNN performed adequately with low-dimensional inputs (3–4 features, R^2^ = 0.86), but their performance dropped sharply for high-dimensional data (R^2^ ≤ 0.80). Ensemble methods, including the newly introduced MBLERM, demonstrated robust performance across feature sets, with MBLERM achieving R^2^ values of approximately 0.82–0.85. These results highlight that careful selection of spectral features and model type is critical for accurate COD retrieval, with ensemble models—particularly CatBoost—offering superior accuracy while excessive features can compromise performance.

**Table 2:** Optimal feature combinations and COD inversion accuracy indicators for different numbers of features

Number of features	Model	Corresponding feature	RMSE	MAE	MSE	MAPE	R^2^	Bias
3 (120 combinations)	CatBoost	B_3-1_	0.47	0.28	0.22	2.39	0.91	0.002
	XGBoost	B_3-2_	0.63	0.35	0.40	3.05	0.85	0.03
	LightGBM	B_3-3_	0.56	0.28	0.32	2.34	0.88	-0.03
	KNN	B_3-4_	0.60	0.20	0.36	1.95	0.86	0.01
	RF	B_3-5_	0.91	0.27	0.83	2.19	0.68	-0.09
	MBLERM	B_3-6_	0.65	0.34	0.43	2.91	0.84	-0.09
4 (210 combinations)	CatBoost	B_4-1_	0.47	0.25	0.22	2.17	0.92	0.02
	XGBoost	B_4-2_	0.64	0.35	0.41	3.03	0.84	0.02
	LightGBM	B_4-3_	0.57	0.30	0.33	2.55	0.87	0.01
	KNN	B_4-4_	0.60	0.17	0.36	1.73	0.86	0.02
	RF	B_4-5_	0.55	0.24	0.30	2.03	0.88	-0.07
	MBLERM	B_4-6_	0.65	0.35	0.43	3.11	0.84	0.05
5 (252 combinations)	CatBoost	B_5-1_	0.42	0.25	0.17	2.11	0.93	0.02
	XGBoost	B_5-2_	0.74	0.32	0.55	2.92	0.79	0.06
	LightGBM	B_5-3_	0.56	0.27	0.32	2.24	0.88	-0.02
	KNN	B_5-4_	0.74	0.14	0.55	1.43	0.79	0.07
	RF	B_5-5_	0.64	0.27	0.41	2.25	0.84	-0.05
	MBLERM	B_5-6_	0.66	0.36	0.43	3.12	0.83	-0.03
6 (210 combinations)	CatBoost	B_6-1_	0.44	0.23	0.19	1.92	0.93	0.01
	XGBoost	B_6-2_	0.63	0.32	0.40	2.83	0.85	0.05
	LightGBM	B_6-3_	0.61	0.31	0.37	2.62	0.86	-0.04
	KNN	B_6-4_	0.71	0.19	0.50	1.84	0.81	0.04
	RF	B_6-5_	0.60	0.25	0.36	2.17	0.86	-0.01
	MBLERM	B_6-6_	0.67	0.42	0.45	3.64	0.83	-0.01
7 (120 combinations)	CatBoost	B_7-1_	0.42	0.22	0.18	1.91	0.93	0.02
	XGBoost	B_7-2_	0.67	0.32	0.45	2.82	0.83	0.06
	LightGBM	B_7-3_	0.64	0.31	0.40	2.66	0.85	-0.03
	KNN	B_7-4_	0.85	0.43	0.73	3.65	0.72	-0.01
	RF	B_7-5_	0.72	0.27	0.51	2.28	0.80	-0.04
	MBLERM	B_7-6_	0.62	0.35	0.39	3.08	0.85	0.07
8 (45 combinations)	CatBoost	B_8-1_	0.48	0.24	0.23	2.06	0.91	0.01
	XGBoost	B_8-2_	0.75	0.35	0.56	3.14	0.79	0.04
	LightGBM	B_8-3_	0.60	0.30	0.36	2.72	0.86	0.06
	KNN	B_8-4_	0.85	0.43	0.71	3.62	0.73	-0.01
	RF	B_8-5_	0.72	0.27	0.52	2.30	0.80	-0.04
	MBLERM	B_8-6_	0.62	0.35	0.39	3.14	0.85	0.03
9 (10 combinations)	CatBoost	B_9-1_	0.48	0.25	0.23	2.23	0.91	0.01
	XGBoost	B_9-2_	0.79	0.38	0.62	3.41	0.76	0.08
	LightGBM	B_9-3_	0.66	0.33	0.43	2.99	0.84	0.06
	KNN	B_9-4_	0.90	0.48	0.80	4.06	0.69	0.02
	RF	B_9-5_	0.78	0.30	0.61	2.60	0.77	-0.04
	MBLERM	B_9-6_	0.69	0.36	0.47	3.14	0.82	0.00

In the table, the corresponding optimal features are as follows: B_3-1_ = [‘*X*3’, ‘*X*6’, ‘*X*9’], B_3-2_ = [‘*X*3’, ‘*X*5’, ‘*X*6’],B_3-3_ = [‘*X*1’, ‘*X*6’, ‘*X*7’], B_3-4_ = [‘*X*2’, ‘*X*3’, ‘*X*10’], B_3-5_ = [‘*X*1’, ‘*X*6’, ‘*X*9’], B_3-6_ = [‘*X*1’, ‘*X*6’, ‘*X*7’], B_4-1_ = [‘*X*2’, ‘*X*3’, ‘*X*5’, ‘*X*6’], B_4-2_ = [‘*X*2’, ‘*X*6’, ‘*X*7’, ‘*X*8’], B_4-3_ = [‘*X*3’, ‘*X*6’, ‘*X*7’, ‘*X*9’], B_4-4_ = [‘*X*1’, ‘*X*2’, ‘*X*3’, ‘*X*10’], B_4-5_ = [‘*X*3’, ‘*X*5’, ‘*X*6’, ‘*X*7’], B_4-6_ = [‘*X*3’, ‘*X*6’, ‘*X*8’, ‘*X*10’], B_5-1_ = [‘*X*3’, ‘*X*5’, ‘*X*6’, ‘*X*7’, ‘*X*9’], B_5-2_ = [‘*X*1’, ‘*X*2’, ‘*X*6’, ‘*X*7’, ‘*X*9’], B_5-3_ = [‘*X*1’, ‘*X*6’, ‘*X*7’, ‘*X*9’, ‘*X*10’], B_5-4_ = [‘*X*1’, ‘*X*3’, ‘*X*4’, ‘*X*6’, ‘*X*10’], B_5-5_ = [‘*X*1’, ‘*X*3’, ‘*X*5’, ‘*X*6’, ‘*X*10’], B_5-6_ = [‘*X*1’, ‘*X*5’, ‘*X*6’, ‘*X*7’, ‘*X*8’], B_6-1_ = [‘*X*1’, ‘*X*3’, ‘*X*6’, ‘*X*7’, ‘*X*8’, ‘*X*10’], B_6-2_ = [‘*X*2’, ‘*X*3’, ‘*X*5’, ‘*X*6’, ‘*X*7’, ‘*X*10’], B_6-3_ = [‘*X*1’, ‘*X*5’, ‘*X*6’, ‘*X*7’, ‘*X*8’, ‘*X*9’], B_6-4_ = [‘*X*1’, ‘*X*2’, ‘*X*3’, ‘*X*4’, ‘*X*6’, ‘*X*10’], B_6-5_ = [‘*X*1’, ‘*X*2’, ‘*X*3’, ‘*X*6’, ‘*X*7’, ‘*X*10’], B_6-6_ = [‘*X*3’, ‘*X*5’, ‘*X*7’, ‘*X*8’, ‘*X*9’, ‘*X*10’], B_7-1_ = [‘*X*1’, ‘*X*3’, ‘*X*4’, ‘*X*6’, ‘*X*7’, ‘*X*8’, ‘*X*10’], B_7-2_ = [‘*X*2’, ‘*X*3’, ‘*X*4’, ‘*X*6’, ‘*X*7’, ‘*X*8’, ‘*X*10’], B_7-3_ = [‘*X*1’, ‘*X*5’, ‘*X*6’, ‘*X*7’, ‘*X*8’, ‘*X*9’, ‘*X*10’], B_7-4_ = [‘*X*1’, ‘*X*2’, ‘*X*3’, ‘*X*4’, ‘*X*6’, ‘*X*7’, ‘*X*9’], B_7-5_ = [‘*X*1’, ‘*X*2’, ‘*X*3’, ‘*X*5’, ‘*X*6’, ‘*X*8’, ‘*X*10’], B_7-6_ = [‘*X*2’, ‘*X*3’, ‘*X*4’, ‘*X*5’, ‘*X*6’, ‘*X*8’, ‘*X*10’], B_8-1_ = [‘*X*1’, ‘*X*2’, ‘*X*3’, ‘*X*4’, ‘*X*6’, ‘*X*7’, ‘*X*8’, ‘*X*10’], B_8-2_ = [‘*X*1’, ‘*X*2’, ‘*X*3’, ‘*X*6’, ‘*X*7’, ‘*X*8’, ‘*X*9’, ‘*X*10’], B_8-3_ = [‘*X*2’, ‘*X*3’, ‘*X*4’, ‘*X*6’, ‘*X*7’, ‘*X*8’, ‘*X*9’, ‘*X*10’], B_8-4_ = [‘*X*1’, ‘*X*2’, ‘*X*3’, ‘*X*4’, ‘*X*6’, ‘*X*7’, ‘*X*9’, ‘*X*10’], B_8-5_ = [‘*X*1’, ‘*X*2’, ‘*X*3’, ‘*X*5’, ‘*X*6’, ‘*X*8’, ‘*X*9’, ‘*X*10’], B_8-6_ = [‘*X*2’, ‘*X*3’, ‘*X*4’, ‘*X*5’, ‘*X*6’, ‘*X*8’, ‘*X*9’, ‘*X*10’], B_9-1_ = [‘*X*1’, ‘*X*2’, ‘*X*3’, ‘*X*4’, ‘*X*5’, ‘*X*6’, ‘*X*7’, ‘*X*8’, ‘*X*10’], B_9-2_ = [‘*X*1’, ‘*X*2’, ‘*X*3’, ‘*X*4’, ‘*X*6’, ‘*X*7’, ‘*X*8’, ‘*X*9’, ‘*X*10’], B_9-3_ = [‘*X*2’, ‘*X*3’, ‘*X*4’, ‘*X*5’, ‘*X*6’, ‘*X*7’, ‘*X*8’, ‘*X*9’, ‘*X*10’], B_9-4_ = [‘*X*1’, ‘*X*2’, ‘*X*3’, ‘*X*4’, ‘*X*6’, ‘*X*7’, ‘*X*8’, ‘*X*9’, ‘*X*10’], B_9-5_ = [‘*X*1’, ‘*X*2’, ‘*X*3’, ‘*X*5’, ‘*X*6’, ‘*X*7’, ‘*X*8’, ‘*X*9’, ‘*X*10’], and B_9-6_ = [‘*X*2’, ‘*X*3’, ‘*X*4’, ‘*X*5’, ‘*X*6’, ‘*X*7’, ‘*X*8’, ‘*X*9’, ‘*X*10’].

**Figure 2:**
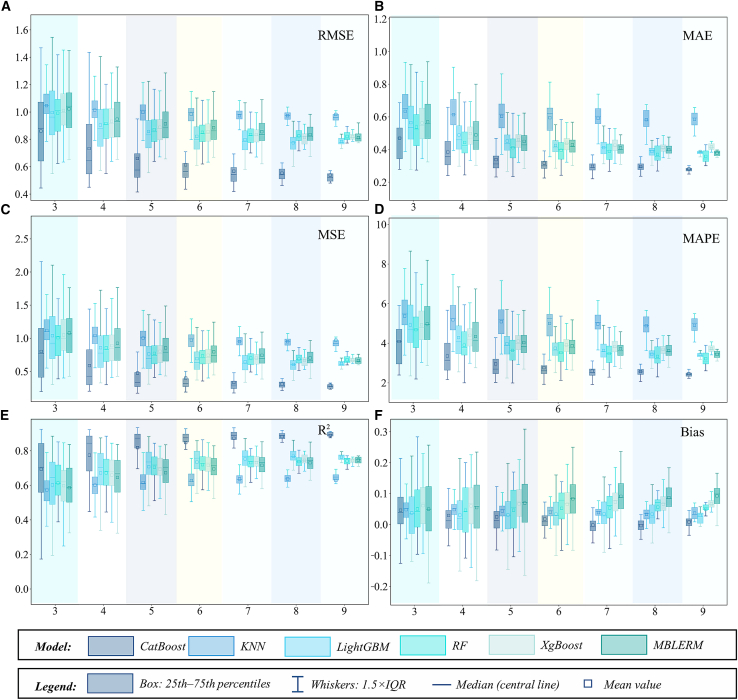
Boxplots of the inversion results accuracy for the COD test set using five algorithms at different feature quantities (A) Boxplots of RMSE. (B) Boxplots of MAE. (C) Boxplots of MSE. (D) Boxplots of MAPE. (E) Boxplots of R^2^. (F) Boxplots of bias.

This study evaluated the COD inversion performance of six machine learning algorithms—CatBoost, XGBoost, LightGBM, KNN, RF, and MBLERM—across varying numbers of spectral features using box plots (Figure 2). CatBoost consistently achieved the best performance, with the mean RMSE decreasing from 0.86 at three features to 0.54 at nine features and the median mean absolute error (MAE) from 0.47 to 0.28, demonstrating superior accuracy and stability, with only minor fluctuations in bias under high-dimensional inputs. MBLERM showed robust performance, achieving moderate-to-high values in MAE, mean absolute percentage Error (MAPE), and R^2^, forming a second tier alongside LightGBM, RF, and XGBoost, and substantially outperforming KNN. Overall, increasing the number of features generally improved accuracy, reduced MAPE, and increased R^2^ for all models, with ensemble methods exhibiting the most pronounced gains. Bias analysis indicated that XGBoost tended to systematically overestimate, whereas the other ensemble models maintained moderate bias. These results highlight the critical importance of careful feature and model selection for accurate COD inversion, with CatBoost representing the optimal choice, while additional features beyond seven provide diminishing returns.

**Figure 3:**
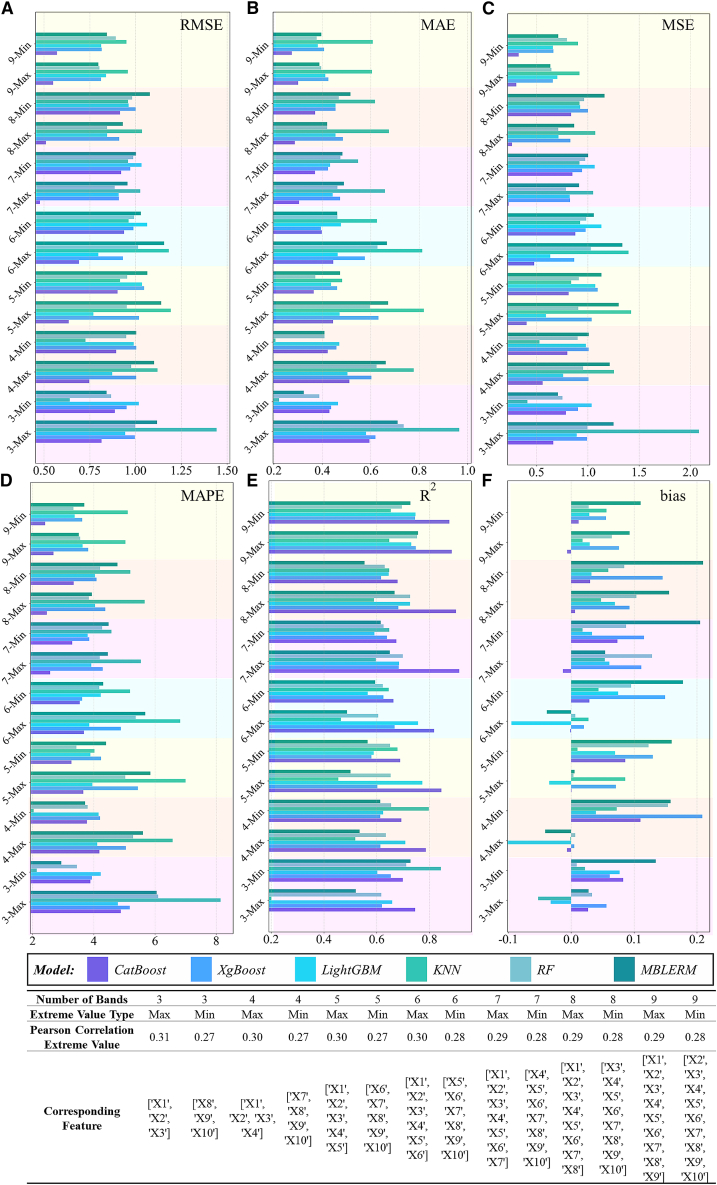
Relationship between $\bar{M}$_*PC*_ and COD model performance across different feature combinations (A) The relationship between $\bar{M}$_PC_ and RMSE. (B) The relationship between $\bar{M}$_PC_ and MAE. (C) The relationship between $\bar{M}$_PC_ and MSE. (D) The relationship between $\bar{M}$_PC_ and MAPE. (E) The relationship between $\bar{M}$_PC_ and R^2^. (F) The relationship between $\bar{M}$_PC_ and bias.

Figure 3 illustrates the relationship between $\bar{M}$_*PC*_ and model performance across different feature combinations. CatBoost consistently achieved the best and most stable performance under both high- and low-correlation features, with the lowest RMSE, mean squared error (MSE), and MAPE observed in the 7-Min combination and R^2^ remaining high. XGBoost and LightGBM showed weaker performance under high-correlation features, while KNN and RF exhibited significantly increased errors under low-correlation features. MBLERM performed robustly across all combinations, with intermediate performance across all metrics, forming a second tier alongside LightGBM, RF, and XGBoost. KNN performed best in high-correlation models with 3–4 features, but its performance fluctuated markedly as the number of features increased. Bias analysis revealed that RF and KNN achieved the lowest bias in the 3-Max combination, whereas CatBoost maintained the most stable bias in high-dimensional combinations; under low-correlation conditions, CatBoost achieved optimal bias control at 6–7 features while maintaining leading overall predictive performance. Notably, the algorithms sometimes exhibited higher accuracy at the lowest $\bar{M}$_*PC*_ than at the mean of the highest correlations; for example, for all algorithms, the 3-Min combination outperformed 3-Max in RMSE, MAE, MSE, MAPE, and R^2^. COD retrieval results indicate that while algorithms selecting the highest-correlation features are commonly used in current water quality retrievals, their effectiveness in machine learning models is limited and does not guarantee optimal model performance.

**Figure 4:**
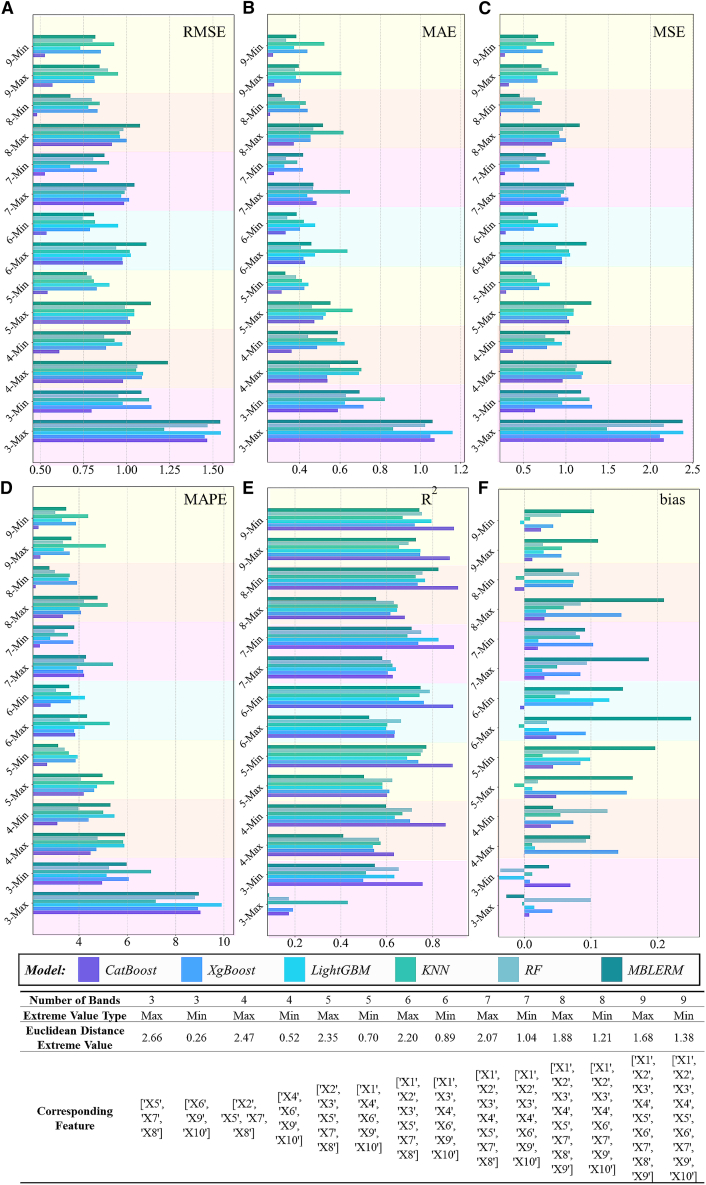
Relationship between $\bar{M}$_*ED*_ and COD model performance across different feature combinations (A) The relationship between $\bar{M}$_ED_ and RMSE. (B) The relationship between $\bar{M}$_ED_ and MAE. (C) The relationship between $\bar{M}$_ED_ and MSE. (D) The relationship between $\bar{M}$_ED_ and MAPE. (E) The relationship between $\bar{M}$_ED_ and R^2^. (F) The relationship between $\bar{M}$_ED_ and bias.

The relationship between the $\bar{M}$_*ED*_ of feature combinations and COD inversion performance indicates that smaller distances generally correspond to lower errors (RMSE, MAE, MSE, and MAPE) and higher R^2^ (Figure 4). CatBoost consistently achieved the best performance, while MBLERM maintained robust secondary accuracy, forming a second tier with LightGBM, RF, and XGBoost. KNN and RF exhibited higher errors at larger distances. Bias was largely unaffected by distance. These results suggest that a more compact feature space enhances predictive accuracy, with $\bar{M}$_*ED*_ serving as a reliable metric for model evaluation.

**Figure 5:**
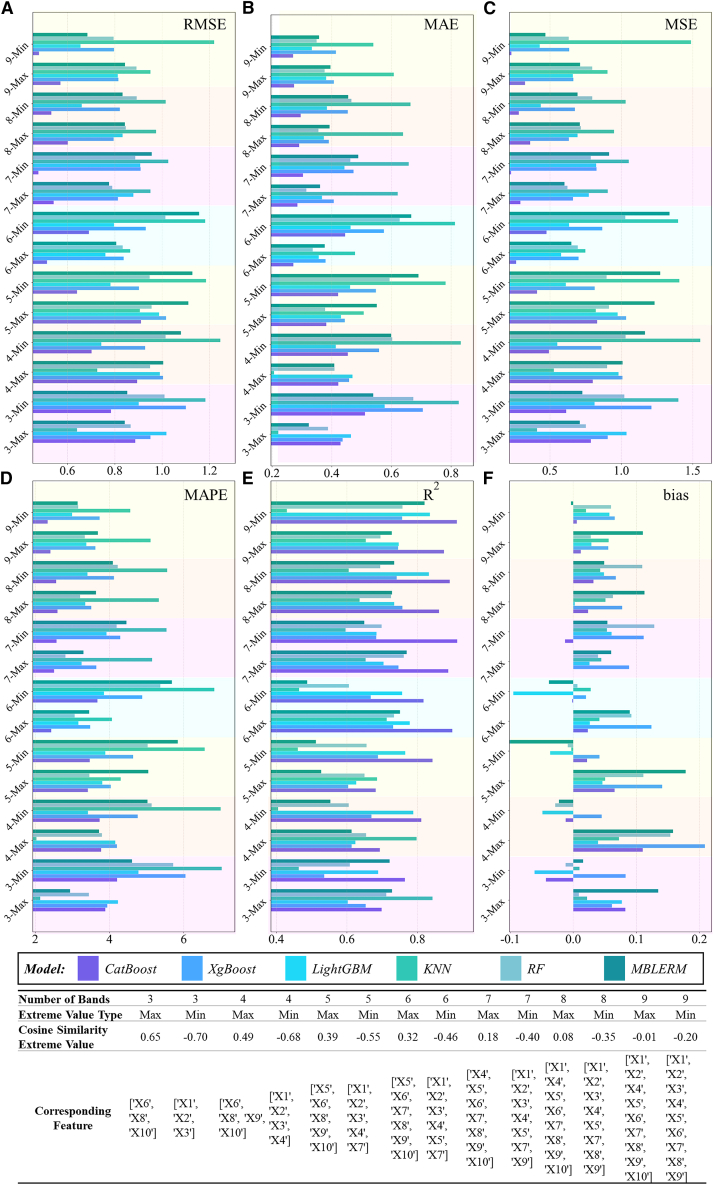
Relationship between $\bar{M}$_CS_ and COD model performance across different feature combinations (A) The relationship between $\bar{M}$_CS_ and RMSE. (B) The relationship between $\bar{M}$_CS_ and MAE. (C) The relationship between $\bar{M}$_CS_ and MSE. (D) The relationship between $\bar{M}$_CS_ and MAPE. (E) The relationship between $\bar{M}$_CS_ and R^2^. (F) The relationship between $\bar{M}$_CS_ and bias.

The $\bar{M}$_*CS*_ analysis for COD inversion (Figure 5) indicates that CatBoost, XGBoost, LightGBM, RF, and MBLERM generally perform better when cosine similarity is higher, with lower RMSE, MAE, MSE, and MAPE and higher R^2^, whereas KNN exhibits the opposite trend, achieving lower errors at lower cosine similarity. Bias values are small across all models, with no systematic bias observed. These findings suggest that most models achieve optimal performance when feature vectors are highly similar, while KNN may be relatively more robust under larger vector differences. Overall, MBLERM demonstrates stable secondary performance, forming a second tier alongside LightGBM, RF, and XGBoost, trailing only the consistently superior CatBoost.

**Figure 6:**
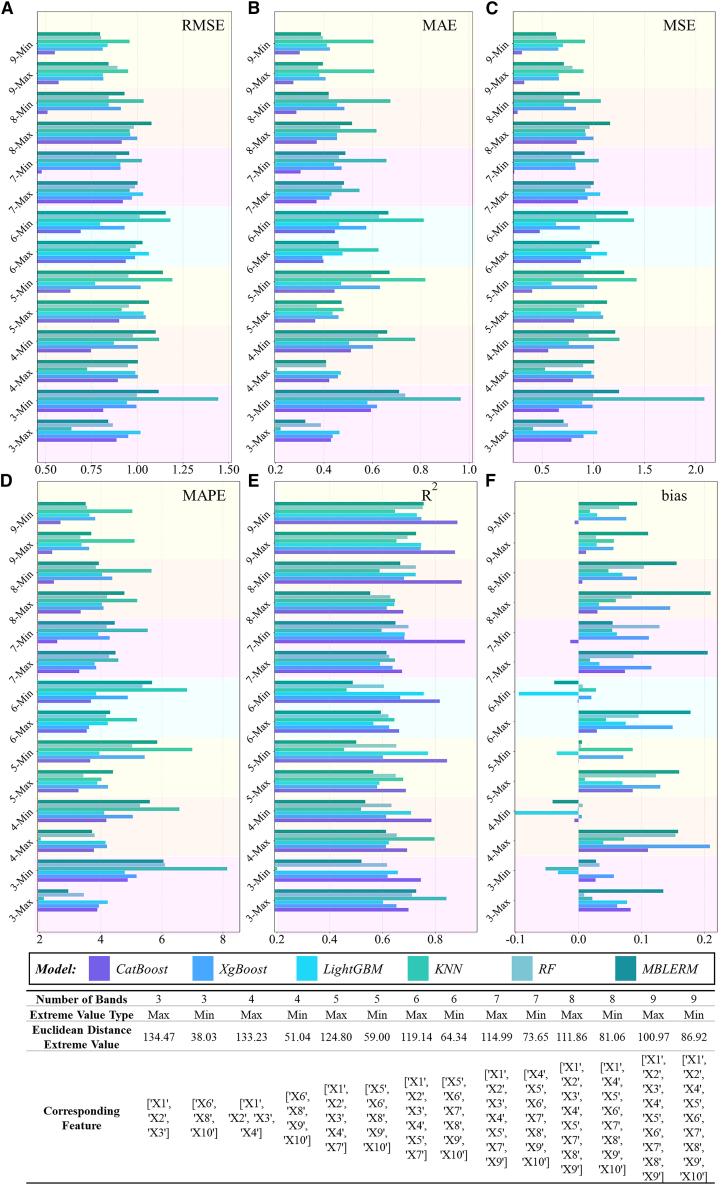
Relationship between $\bar{M}$_*SA*_ and COD model performance across different feature combinations (A) The relationship between $\bar{M}$_SA_ and RMSE. (B) The relationship between $\bar{M}$_SA_ and MAE. (C) The relationship between $\bar{M}$_SA_ and MSE. (D) The relationship between $\bar{M}$_SA_ and MAPE. (E) The relationship between $\bar{M}$_SA_ and R^2^. (F) The relationship between $\bar{M}$_SA_ and bias.

The $\bar{M}$_*SA*_ and COD inversion performance (Figure 6) indicates that, except for KNN, most models achieve lower errors (RMSE, MAE, MSE, and MAPE) and higher R^2^ at smaller spectral angles. In contrast, KNN exhibits the opposite trend across multiple feature counts, with lower errors occurring at larger spectral angles. For example, at five features, CatBoost attains an RMSE of 0.64 at the minimum spectral angle compared with 0.90 at the maximum, whereas KNN shows 1.19 at the minimum and 0.92 at the maximum spectral angle. Rankings based on RMSE, MAE, MSE, and MAPE are generally consistent and align with R^2^. Bias values are overall small, although noticeable positive or negative deviations occur in a few model/feature combinations.

### Impact of feature combinations on the accuracy of NH_3_-N retrieval in aquatic environments

For NH_3_-N concentration inversion, we applied the same modeling methods and scale as for COD, utilizing six machine learning algorithms. A total of 5,802 models were developed based on all possible combinations of features. Table 3 shows that model performance varied dynamically with the dimensionality of the feature space. CatBoost performed best at low dimensionality (3 features), while RF outperformed other models at 4–6 features. As the dimensionality increased to 7 features, XGBoost demonstrated optimal performance. The MBLERM model exhibited excellent stability and competitiveness across all feature dimensions, achieving robust results in RMSE, MAE, and MSE. Compared with other models, MBLERM showed outstanding performance, especially at higher feature dimensionalities, demonstrating its strong capabilities. Optimal feature combinations frequently included features *X*4, *X*8, and *X*9, highlighting their crucial role in NH_3_-N inversion. Model performance generally improved initially and then deteriorated as the number of features increased. When the feature count exceeded seven, accuracy generally declined, with the KNN model experiencing a performance collapse at nine features, confirming the presence of the curse of dimensionality. These findings provide valuable insights into feature selection and model optimization for water quality parameter inversion.

**Table 3:** Optimal feature combinations and NH3-N inversion accuracy indicators for different numbers of features

Number of feature	Model	Features	RMSE	MAE	MSE	MAPE	R^2^	Bias
3 (120 combinations)	CatBoost	B_3-1_	0.01	0.006	0.0002	1.44	0.9998	0.0001
	XGBoost	B_3-2_	0.03	0.014	0.0011	3.18	0.9985	0.0032
	LightGBM	B_3-3_	0.02	0.010	0.0002	2.59	0.9997	−0.0041
	KNN	B_3-4_	0.02	0.0088	0.0005	2.25	0.9993	−0.0009
	RF	B_3-5_	0.03	0.004	0.0006	0.78	0.9991	−0.0039
	MBLERM	B_3-6_	0.02	0.014	0.0004	3.33	0.9994	0.0011
4 (210 combinations)	CatBoost	B_4-1_	0.02	0.010	0.0004	2.17	0.9994	−0.0018
	XGBoost	B_4-2_	0.03	0.014	0.0008	3.25	0.9988	0.0029
	LightGBM	B_4-3_	0.02	0.011	0.0003	2.75	0.9996	0.0001
	KNN	B_4-4_	0.02	0.004	0.0003	1.23	0.9995	0.0016
	RF	B_4-5_	0.01	0.003	0.0002	0.81	0.9998	−0.0022
	MBLERM	B_4-6_	0.02	0.011	0.0002	2.42	0.9996	−0.0022
5 (252 combinations)	CatBoost	B_5-1_	0.03	0.013	0.0011	2.92	0.9985	−0.0022
	XGBoost	B_5-2_	0.02	0.009	0.0003	2.12	0.9996	−0.0023
	LightGBM	B_5-3_	0.02	0.015	0.0004	3.73	0.9994	−0.0048
	KNN	B_5-4_	0.02	0.004	0.0003	1.01	0.9996	0.0016
	RF	B_5-5_	0.01	0.002	0.0001	0.59	0.9999	0.0018
	MBLERM	B_5-6_	0.03	0.015	0.0009	2.81	0.9988	0.0012
6 (210 combinations)	CatBoost	B_6-1_	0.03	0.018	0.0008	4.04	0.9988	0.0070
	XGBoost	B_6-2_	0.03	0.012	0.0007	2.71	0.9991	−0.0032
	LightGBM	B_6-3_	0.02	0.018	0.0005	3.99	0.9992	−0.0038
	KNN	B_6-4_	0.02	0.005	0.0003	1.33	0.9996	0.0013
	RF	B_6-5_	0.01	0.002	0.0001	0.56	0.9999	0.0009
	MBLERM	B_6-6_	0.03	0.018	0.0011	3.36	0.9984	−0.0065
7 (120 combinations)	CatBoost	B_7-1_	0.03	0.012	0.0012	3.05	0.9984	0.0067
	XgBoost	B_7-2_	0.02	0.009	0.0004	2.40	0.9995	0.0016
	LightGBM	B_7-3_	0.04	0.021	0.0013	4.66	0.9981	−0.0017
	KNN	B_7-4_	0.03	0.006	0.0008	1.63	0.9989	0.0021
	RF	B_7-5_	0.03	0.007	0.0010	1.47	0.9986	−0.0022
	MBLERM	B_7-6_	0.03	0.020	0.0011	3.73	0.9984	0.0019
8 (45 combinations)	CatBoost	B_8-1_	0.05	0.028	0.0030	5.68	0.9958	0.0031
	XGBoost	B_8-2_	0.05	0.021	0.0021	4.42	0.9970	0.0047
	LightGBM	B_8-3_	0.06	0.025	0.0036	5.14	0.9949	−0.0129
	KNN	B_8-4_	0.03	0.008	0.0009	2.30	0.9988	0.0034
	RF	B_8-5_	0.04	0.012	0.0018	2.42	0.9975	−0.0082
	MBLERM	B_8-6_	0.03	0.019	0.0009	4.02	0.9988	0.0024
9 (10 combinations)	CatBoost	B_9-1_	0.07	0.034	0.0050	7.32	0.9929	0.0080
	XGBoost	B_9-2_	0.11	0.033	0.0123	4.89	0.9826	−0.0129
	LightGBM	B_9-3_	0.06	0.026	0.0041	5.36	0.9942	−0.0124
	KNN	B_9-4_	0.52	0.14	0.265	19.64	0.6232	0.0808
	RF	B_9-5_	0.04	0.015	0.0017	3.16	0.9975	0.0083
	MBLERM	B_9-6_	0.11	0.029	0.0114	5.79	0.9838	0.0033

In the table, the corresponding optimal features are as follows: B_3-1_ = [‘*X*1’, ‘*X*4’, ‘*X*9’], B_3-2_ = [‘*X*4’, ‘*X*8’, ‘*X*9’], B_3-3_ = [‘*X*2’, ‘*X*8’, ‘*X*9’], B_3-4_ = [‘*X*5’, ‘*X*8’, ‘*X*10’], B_3-5_ = [‘*X*5’, ‘*X*9’, ‘*X*10’], B_3-6_ = [‘*X*4’, ‘*X*5’, ‘*X*9’], B_4-1_ = [‘*X*1’, ‘*X*4’, ‘*X*9’, ‘*X*10’], B_4-2_ = [‘*X*1’, ‘*X*4’, ‘*X*8’, ‘*X*10’], B_4-3_ = [‘*X*2’, ‘*X*7’, ‘*X*8’, ‘*X*9’], B_4-4_ = [‘*X*4’, ‘*X*5’, ‘*X*8’, ‘*X*10’], B_4-5_ = [‘*X*5’, ‘*X*6’, ‘*X*8’, ‘*X*9’], B_4-6_ = [‘*X*1’, ‘*X*2’, ‘*X*6’, ‘*X*10’], B_5-1_ = [‘*X*4’, ‘*X*7’, ‘*X*8’, ‘*X*9’, ‘*X*10’], B_5-2_ = [‘*X*1’, ‘*X*4’, ‘*X*7’, ‘*X*8’, ‘*X*9’], B_5-3_ = [‘*X*2’, ‘*X*6’, ‘*X*7’, ‘*X*8’, ‘*X*9’], B_5-4_ = [‘*X*4’, ‘*X*5’, ‘*X*8’, ‘*X*9’, ‘*X*10’], B_5-5_ = [‘*X*1’, ‘*X*4’, ‘*X*5’, ‘*X*8’, ‘*X*9’], B_5-6_ = [‘*X*1’, ‘*X*4’, ‘*X*5’, ‘*X*8’, ‘*X*10’], B_6-1_ = [‘*X*2’, ‘*X*4’, ‘*X*6’, ‘*X*7’, ‘*X*8’, ‘*X*9’], B_6-2_ = [‘*X*4’, ‘*X*6’, ‘*X*7’, ‘*X*8’, ‘*X*9’, ‘*X*10’], B_6-3_ = [‘*X*2’, ‘*X*5’, ‘*X*6’, ‘*X*7’, ‘*X*8’, ‘*X*9’], B_6-4_ = [‘*X*4’, ‘*X*5’, ‘*X*7’, ‘*X*8’, ‘*X*9’, ‘*X*10’], B_6-5_ = [‘*X*1’, ‘*X*4’, ‘*X*5’, ‘*X*6’, ‘*X*8’, ‘*X*9’], B_6-6_ = [‘*X*4’, ‘*X*5’, ‘*X*7’, ‘*X*8’, ‘*X*9’, ‘*X*10’], B_7-1_ = [‘*X*2’, ‘*X*3’, ‘*X*5’, ‘*X*6’, ‘*X*7’, ‘*X*9’, ‘*X*10’], B_7-2_ = [‘*X*2’, ‘*X*4’, ‘*X*5’, ‘*X*7’, ‘*X*8’, ‘*X*9’, ‘*X*10’], B_7-3_ = [‘*X*1’, ‘*X*2’, ‘*X*5’, ‘*X*7’, ‘*X*8’, ‘*X*9’, ‘*X*10’], B_7-4_ = [‘*X*3’, ‘*X*4’, ‘*X*5’, ‘*X*7’, ‘*X*8’, ‘*X*9’, ‘*X*10’], B_7-5_ = [‘*X*1’, ‘*X*4’, ‘*X*5’, ‘*X*6’, ‘*X*8’, ‘*X*9’, ‘*X*10’], B_7-6_ = [‘*X*1’, ‘*X*4’, ‘*X*5’, ‘*X*6’, ‘*X*7’, ‘*X*9’, ‘*X*10’], B_8-1_ = [‘*X*1’, ‘*X*2’, ‘*X*3’, ‘*X*4’, ‘*X*6’, ‘*X*8’, ‘*X*9’, ‘*X*10’], B_8-2_ = [‘*X*2’, ‘*X*3’, ‘*X*5’, ‘*X*6’, ‘*X*7’, ‘*X*8’, ‘*X*9’, ‘*X*10’], B_8-3_ = [‘*X*1’, ‘*X*2’, ‘*X*4’, ‘*X*5’, ‘*X*6’, ‘*X*7’, ‘*X*9’, ‘*X*10’], B_8-4_ = [‘*X*3’, ‘*X*4’, ‘*X*5’, ‘*X*6’, ‘*X*7’, ‘*X*8’, ‘*X*9’, ‘*X*10’], B_8-5_ = [‘*X*1’, ‘*X*4’, ‘*X*5’, ‘*X*6’, ‘*X*7’, ‘*X*8’, ‘*X*9’, ‘*X*10’], B_8-6_ = [‘*X*2’, ‘*X*4’, ‘*X*5’, ‘*X*6’, ‘*X*7’, ‘*X*8’, ‘*X*9’, ‘*X*10’], B_9-1_ = [‘*X*1’, ‘*X*2’, ‘*X*3’, ‘*X*4’, ‘*X*5’, ‘*X*7’, ‘*X*8’, ‘*X*9’, ‘*X*10’], B_9-2_ = [‘*X*1’, ‘*X*2’, ‘*X*4’, ‘*X*5’, ‘*X*6’, ‘*X*7’, ‘*X*8’, ‘*X*9’, ‘*X*10’], B_9-3_ = [‘*X*1’, ‘*X*2’, ‘*X*4’, ‘*X*5’, ‘*X*6’, ‘*X*7’, ‘*X*8’, ‘*X*9’, ‘*X*10’], B_9-4_ = [‘*X*1’, ‘*X*3’, ‘*X*4’, ‘*X*5’, ‘*X*6’, ‘*X*7’, ‘*X*8’, ‘*X*9’, ‘*X*10’], B_9-5_ = [‘*X*1’, ‘*X*3’, ‘*X*4’, ‘*X*5’, ‘*X*6’, ‘*X*7’, ‘*X*8’, ‘*X*9’, ‘*X*10’], and B_9-6_ = [‘*X*1’, ‘*X*3’, ‘*X*4’, ‘*X*5’, ‘*X*6’, ‘*X*7’, ‘*X*8’, ‘*X*9’, ‘*X*10’].

**Figure 7:**
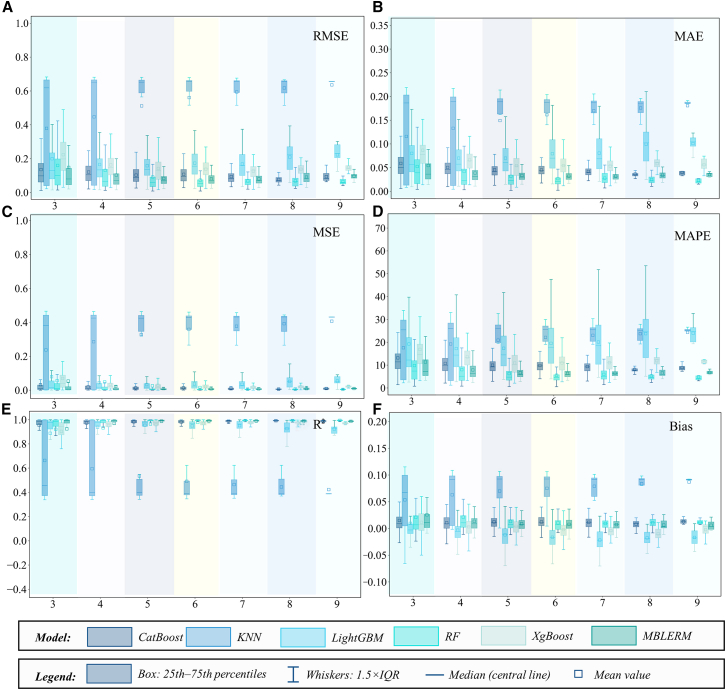
Boxplots of the inversion results accuracy for the NH_3_-N test set using five algorithms at different feature quantities (A) Boxplots of RMSE. (B) Boxplots of MAE. (C) Boxplots of MSE. (D) Boxplots of MAPE. (E) Boxplots of R^2^. (F) Boxplots of bias.

Figure 7 presents the performance of six models on the NH_3_-N test set across varying feature numbers (3–9). Overall, the RMSE median and interquartile range (IQR) of most models decrease slightly, with an increase in feature numbers. KNN is the most unstable at 3 features and tends to overfit at higher feature counts. RF remains stable throughout, achieving the lowest RMSE at 7–9 features, with the smallest mean MAE, a reduction in MAPE from 7.40% to 4.12%, R^2^ consistently above 0.99, and bias close to zero, demonstrating the best overall performance. CatBoost also shows robustness, with its IQR narrowing as the number of features increases, along with excellent performance in MAE, MSE, R^2^, and bias. MBLERM demonstrates solid stability and competitiveness in RMSE, MAE, MSE, and bias. XGBoost exhibits good unbiasedness, while LightGBM shows greater fluctuations and a pronounced negative bias. The NH_3_-N retrieval results similarly confirmed conclusions observed for COD. Under the same number of features, higher correlation did not necessarily lead to higher retrieval accuracy, and most models performed better under low-correlation conditions.

**Figure 8:**
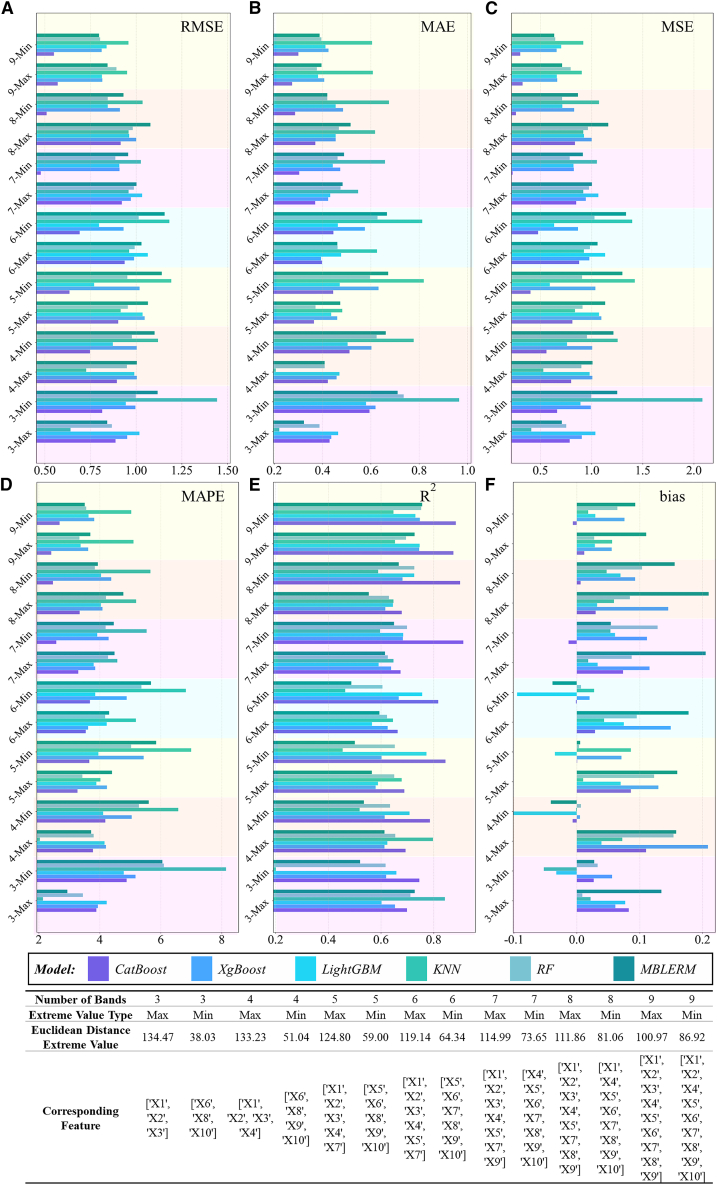
Relationship between $\bar{M}$_*PC*_ and NH3-N model performance across different feature combinations (A) The relationship between $\bar{M}$_PC_ and RMSE. (B) The relationship between $\bar{M}$_PC_ and MAE. (C) The relationship between $\bar{M}$_PC_ and MSE. (D) The relationship between $\bar{M}$_PC_ and MAPE. (E) The relationship between $\bar{M}$_PC_ and R^2^. (F) The relationship between $\bar{M}$_PC_ and bias.

Figure 8 shows the relationship between the performance of NH_3_-N inversion models and the $\bar{M}$_*PC*_ values under different feature combinations. CatBoost consistently outperforms all models in NH_3_-N inversion, while KNN performs well on low-correlation, low-dimensional data but deteriorates with more features. RF and MBLERM exhibit robust stability across feature combinations. MAE and MSE trends mirror RMSE, with KNN, LightGBM, and XGBoost underperforming at high correlation with few features. MAPE is generally higher for small feature numbers, with RF achieving the lowest value at minimal correlation. R^2^ shows that high Pearson correlation does not always imply better fit, and bias remains near zero for all models. Overall, increasing feature numbers improves performance, and CatBoost and MBLERM provide the most reliable inversion.

**Figure 9:**
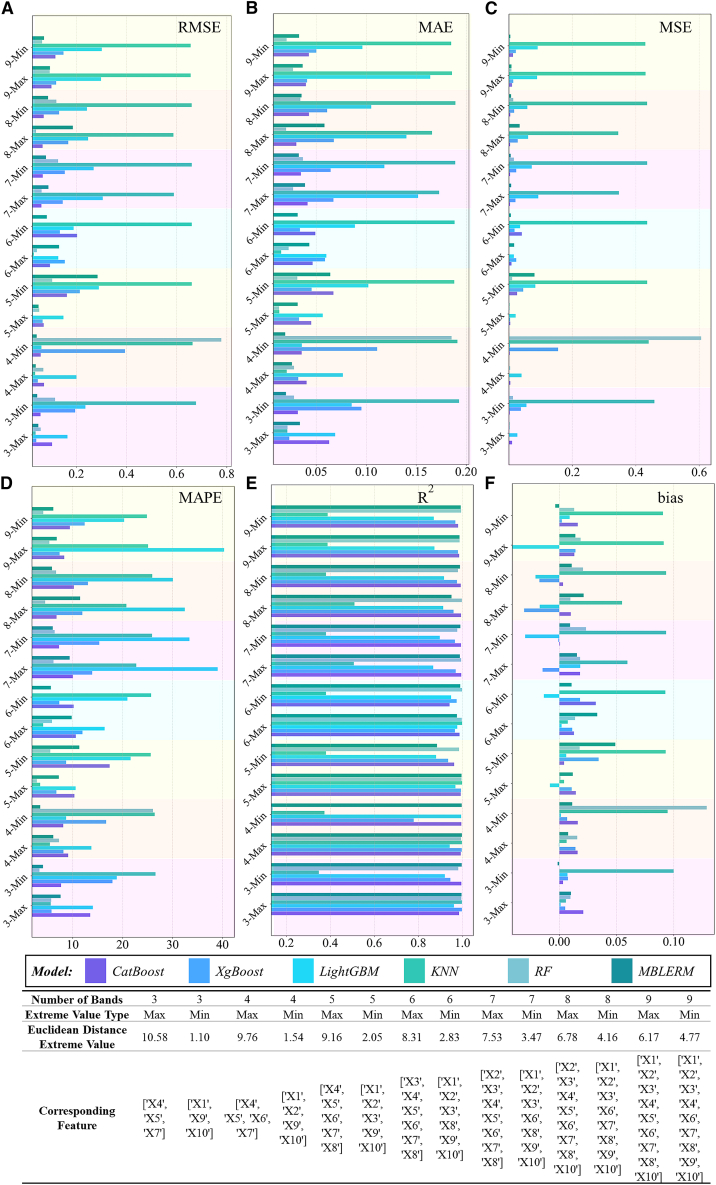
Relationship between $\bar{M}$_*ED*_ and NH_3_-N model performance across different feature combinations (A) The relationship between $\bar{M}$_ED_ and RMSE. (B) The relationship between $\bar{M}$_ED_ and MAE. (C) The relationship between $\bar{M}$_ED_ and MSE. (D) The relationship between $\bar{M}$_ED_ and MAPE. (E) The relationship between $\bar{M}$_ED_ and R^2^. (F) The relationship between $\bar{M}$_ED_ and bias.

Analysis of the $\bar{M}$_*ED*_ across different feature combinations reveals a clear relationship with NH_3_-N inversion accuracy (Figure 9). Unlike COD, NH_3_-N has different dimensional units, resulting in distinct trends. Larger $\bar{M}$_*ED*_ generally correspond to better model performance, including lower RMSE, MAE, MSE, and MAPE, as well as higher R^2^. KNN results are highly variable. As the number of features increases, the influence of $\bar{M}$_*ED*_ on performance gradually stabilizes. These findings indicate that larger $\bar{M}$_*ED*_ enhances NH_3_-N inversion performance, with the robustness of CatBoost and MBLERM ensuring reliable inversion across feature combinations.

**Figure 10:**
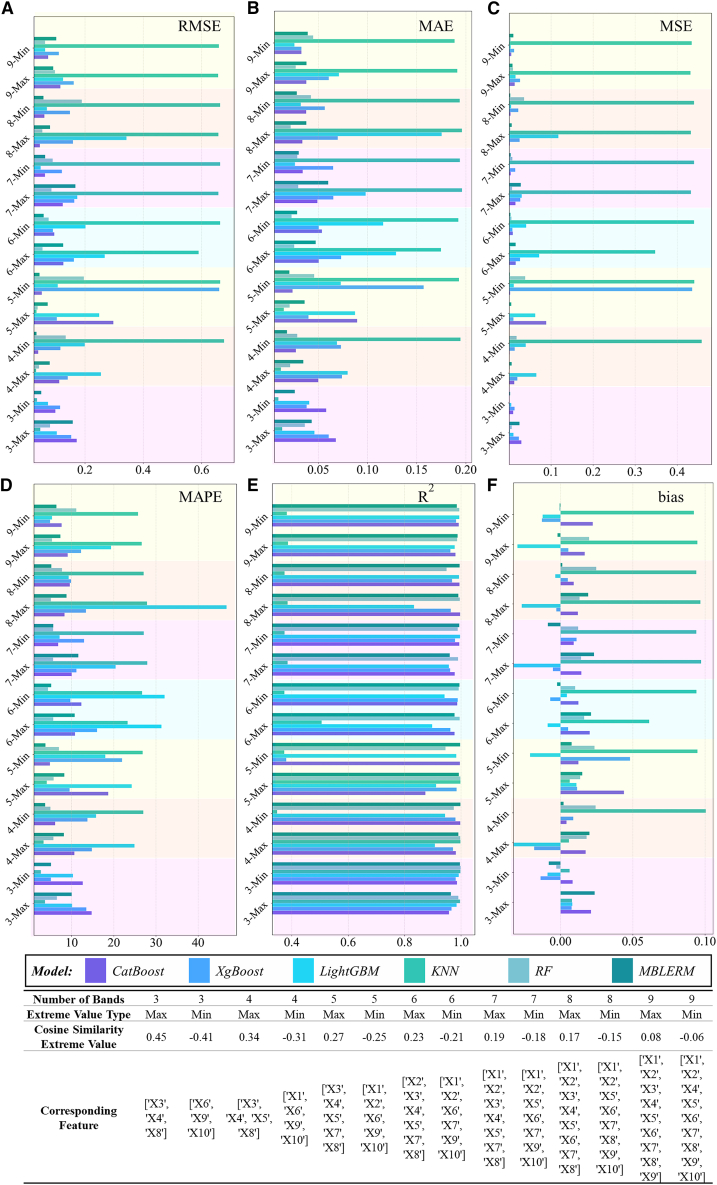
Relationship between $\bar{M}$_*CS*_ and NH_3_-N model performance across different feature combinations (A) The relationship between $\bar{M}$_CS_ and RMSE. (B) The relationship between $\bar{M}$_CS_ and MAE. (C) The relationship between $\bar{M}$_CS_ and MSE. (D) The relationship between $\bar{M}$_CS_ and MAPE. (E) The relationship between $\bar{M}$_CS_ and R^2^. (F) The relationship between $\bar{M}$_CS_ and bias.

The influence of $\bar{M}$_*CS*_ on NH_3_-N inversion across different features (Figure 10) indicates that most models, including CatBoost, XGBoost, LightGBM, RF, and MBLERM, tend to achieve lower errors (RMSE, MAE, MSE, and MAPE) and higher R^2^ in the the Min group. However, for 5- and 8-feature models, XGBoost and RF perform better with the Max group, suggesting that when feature dimensions align with their gain mechanisms, higher cosine similarity can enhance the fit. KNN generally performs better in the Max group, except for three-feature models, consistent with its neighborhood-based mechanism, where more similar vectors (larger cosine, smaller Euclidean distance) improve local prediction. Overall, error-based metrics and goodness-of-fit metrics do not monotonically improve with increasing cosine similarity, indicating that larger cosine similarity does not necessarily guarantee higher accuracy, and there is a significant interaction among model type, feature number, and similarity.

**Figure 11:**
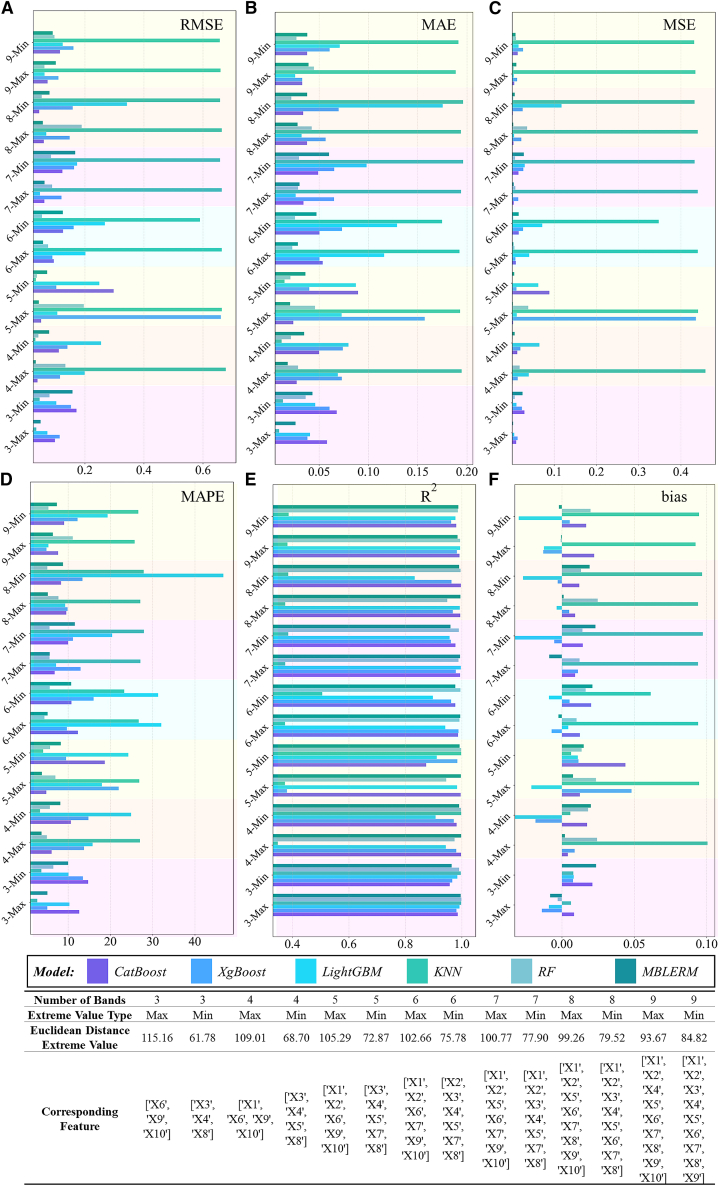
Relationship between $\bar{M}$_*SA*_ and NH_3_-N model performance across different feature combinations (A) The relationship between $\bar{M}$_SA_ and RMSE. (B) The relationship between $\bar{M}$_SA_ and MAE. (C) The relationship between $\bar{M}$_SA_ and MSE. (D) The relationship between $\bar{M}$_SA_ and MAPE. (E) The relationship between $\bar{M}$_SA_ and R^2^. (F) The relationship between $\bar{M}$_SA_ and bias.

Spectral angle $\bar{M}$_*SA*_ and NH_3_-N inversion accuracy (Figure 11) show that for 3–4 features, larger angles (lower similarity) generally yield smaller errors and higher R^2^, e.g., 3-feature RF. For 5–8 features, KNN, RF, CatBoost, and MBLERM perform better at smaller angles, while LightGBM and XGBoost sometimes favor larger angles. For 9 features, most models prefer larger angles. Optimal accuracy is concentrated in specific RF, KNN, CatBoost, and MBLERM combinations, with bias mostly 10^−3^–10^−2^. This indicates that prediction accuracy depends not only on spectral similarity but also on feature information content and model structure: small angles do not always guarantee better accuracy, whereas differentiated spectral shapes linked to NH_3_-N can enhance performance even at larger angles.

### Spatial analysis of water quality inversion results

**Figure 12:**
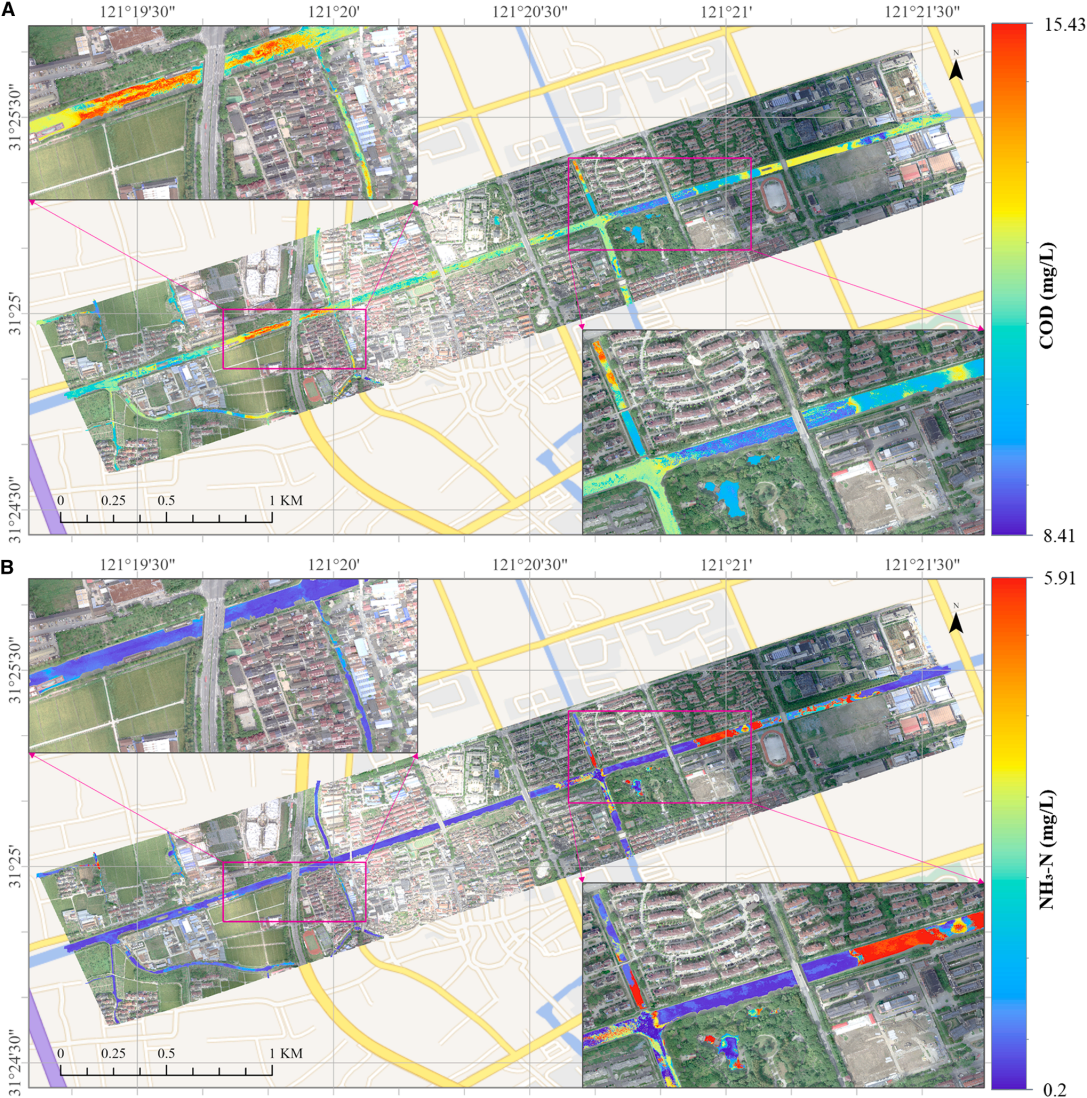
UAV-borne remote sensing water quality inversion results (A) Spatial distribution of COD concentration. (B) Spatial distribution of NH3-N concentration.

To comprehensively reveal the spatial differentiation patterns of the water environment in the study area, this study performs inversions of the imagery based on the optimal accuracy evaluation results from the test set and presents the spatial distribution maps of both parameters (Figure 12). The optimal algorithm for COD inversion is CatBoost, with the optimal features being ['*X*3', '*X*5', '*X*6', '*X*7', '*X*9']. For NH_3_-N inversion, the optimal algorithm is RF, with the corresponding features being ['*X*1', '*X*4', '*X*5', '*X*6', '*X*8', '*X*9']. These two key water quality parameters exhibit significant spatial heterogeneity in the study area. The concentration range of COD is 8.41–15.43 mg/L, with its spatial pattern showing distinct aggregation characteristics. High-concentration areas (>12 mg/L) are mainly distributed along the main rivers in the southwestern part of the study area, surrounded by farmland and residential communities. The concentration range for NH_3_-N is wider (0.2–5.91 mg/L), and its spatial distribution is more complex. High-concentration areas (>3 mg/L) are distributed in a multi-center pattern, primarily in the northeastern part of the study area, where densely populated residential and industrial areas are located. This finding confirms that the water quality in the study area is directly associated with various pollution sources, including industrial, domestic, and agricultural non-point sources. Future studies could integrate hydrological models and pollution source inventories to further quantify the contribution rates of different pollution sources, providing scientific support for the development of differentiated control measures.

## Discussion

### Importance of feature selection

Traditional water quality remote sensing research typically adopts the “high correlation band priority” selection strategy. However, the results of the full combination band analysis in this study reveal that the optimal feature combinations are not necessarily positively correlated with linear correlation, challenging the conventional selection paradigm based on the Pearson correlation coefficient. The underlying reason is that machine learning models, especially ensemble algorithms, can capture nonlinear interactions between features,^58^^,^^59^^,^^60^ and high-correlation features may contain redundant information. The multi-dimensional feature evaluation system proposed in this study (including $\bar{M}$_*PC*_, $\bar{M}$_*ED*_, $\bar{M}$_*CS*_, and $\bar{M}$_*SA*_) incorporates distance metrics, directional similarity, and related indicators, which may provide a tentative or complementary perspective for the quantitative assessment of feature combinations.

However, the feature selection approach employed in this study still presents several limitations: (1) although the exhaustive search strategy for feature combinations ensures a comprehensive exploration, its computational cost grows exponentially with the number of features. This could pose a computational efficiency bottleneck when applied to high-dimensional spectral data. (2) The spatiotemporal coverage of this study is relatively limited. The potential specificity of the proposed feature selection criteria in response to water quality parameters across varying temporal and geographic contexts remains unverified, which somewhat restricts the method’s generalizability. (3) Although this study reveals the critical phenomenon that “high correlation does not equate to high predictive performance” through a full combination analysis and constructs a multi-dimensional feature evaluation system, it lacks a systematic comparison with standard feature selection methods widely adopted in remote sensing water quality inversion (e.g., RFE, SFS, and permutation-based feature importance). This omission leaves the incremental value of the proposed framework and the necessity of its methodological complexity without sufficient empirical support, constituting a clear limitation of the current study.

Therefore, future research should consider comparing this method with the aforementioned mainstream feature selection benchmarks across multiple dimensions under unified experimental settings (including consistent data partitioning, cross-validation strategies, and hyperparameter optimization pipelines). Evaluation metrics could encompass inversion accuracy, model stability, and cross-algorithm generalization capability. Additionally, validation across broader geographic regions and diverse water body types is necessary to verify the universality of these conclusions. Ultimately, integrating the “feature-algorithm synergy” mechanism—as preliminarily revealed in this study—with efficient search strategies, such as Bayesian optimization, holds promise for developing a hybrid feature selection framework that balances global exploration capability with computational efficiency. This advancement would propel remote sensing water quality inversion away from a reliance on empirically derived correlations and toward a new paradigm of intelligent modeling centered on mechanistic understanding and algorithmic adaptation.

### Nonlinear relationship between model performance and the number of spectral features

The selection of an optimal algorithm is highly dependent on the dimensionality of the feature space, and this dependency is further influenced by the type of water quality parameter and the modeling algorithm. In water quality retrieval, model performance does not follow a “more is better” principle with respect to the number of bands. For example, some metrics exhibit a nonlinear trend of initial improvement, followed by saturation or even degradation, reflecting a dynamic balance between information gain and the curse of dimensionality in high-dimensional spaces. When the number of features is relatively low (e.g., 3–7), adding features generally introduces complementary information, significantly enhancing accuracy. However, beyond a certain threshold (7–9 features in this study), the marginal benefits of additional features diminish rapidly, and redundancy and noise begin to dominate, leading to stagnation or decline in performance. Notably, this threshold varies with the target parameter: COD performs optimally with 6–8 features, whereas NH_3_-N favors 4–6 features. In comparison, ensemble learning algorithms (e.g., CatBoost and MBLERM) are more robust to high-dimensional redundancy, exhibiting a more gradual decline in performance. Although the present study identifies these general trends, a quantitative analysis of how redundant information specifically affects the internal mechanisms of different algorithms is still lacking, and the integration of information-theoretic metrics with feature evolution remains to be explored in depth.

Future research will aim to further elucidate these underlying mechanisms by integrating feature importance analysis, information-theoretic approaches, and intelligent optimization techniques. The goal is to quantitatively characterize the evolution of information redundancy and noise during band expansion, and, based on these insights, to develop theoretical and methodological frameworks for the automated identification of optimal feature sets. This approach will provide mechanistic explanations for performance saturation phenomena and facilitate a shift from empirical feature selection toward interpretable, efficient discovery of truly optimal feature combinations.

### Spatial heterogeneity of water quality

The spatial distribution results indicate that areas with high COD values (>12 mg/L) are predominantly located along the main river channels in the southwestern part of the study area, which is mainly characterized by farmland, while areas with high NH_3_-N values (>3 mg/L) are significantly concentrated in the industrial and residential pollution source-rich regions in the central-northern part. This marked spatial differentiation not only aligns closely with the distribution of pollution sources observed in the field but also validates the reliability of the inversion results. Additionally, it reveals the unique spatial response specificity of different water quality parameters to their dominant pollution sources (with COD corresponding to agricultural non-point sources and NH_3_-N corresponding to industrial and residential point sources).^59^^,^^61^ This provides a direct basis for pollution source tracing based on spatial patterns.

However, there are still several limitations in the analysis of spatial heterogeneity in this study: (1) the analysis of the spatial distribution mechanisms remains at a qualitative description level and has not incorporated quantitative driving force analysis of the intrinsic relationship between land use and water quality spatial differentiation, such as using landscape pattern indices or pollution load quantification models; (2) the study only analyzed the spatial patterns of water quality at a single time point and did not account for the seasonal or event-based impacts of dynamic hydrological processes, such as rainfall and runoff, on the spatial distribution of pollutants. This limitation restricts the extrapolation capability of the inversion results in the temporal dimension. Future research should focus on key aspects such as quantitative analysis of spatial driving forces and the coupling of dynamic hydrological processes to enhance the spatial early-warning and dynamic-monitoring capabilities of water quality remote sensing inversion.

In summary, this study explores the inversion performance of machine learning algorithms for urban river water quality parameters (COD and NH3-N) by constructing a total of 5,802 evaluation models under different feature selection schemes. Key findings include: (1) traditional correlation metrics, such as Pearson’s coefficient, are not the sole criterion for feature selection. A multi-dimensional indicator system ($\bar{M}$_*PC*_, $\bar{M}$_*ED*_, $\bar{M}$_*CS*_, and $\bar{M}$_*SA*_) shows that high correlation does not always guarantee high inversion accuracy, with ensemble learning algorithms capturing complex spectral features beyond linear relationships. (2) Model performance exhibits a non-linear relationship with the number of features, with optimal results achieved at 5–7 features for COD and 3–6 features for NH3-N, highlighting the curse of dimensionality and the importance of model selection based on feature space dimensions. (3) Spatial inversions reveal that high-COD concentrations are concentrated in agricultural areas, while NH3-N pollution hotspots are in industrial and residential zones, directly reflecting the impact of different pollution sources on water quality, providing insights for targeted management strategies.

### Limitations of the study

This study has two main limitations. First, the exhaustive search for feature combinations ensures full exploration but incurs exponentially increasing computational costs with feature dimensionality, limiting its efficiency for high-dimensional hyperspectral data. Second, the research is confined to a single temporal snapshot and a single study area (Lianqi River, Shanghai), lacking cross-temporal and cross-water body validation, which restricts the generalizability of the findings.

## Resource availability

### Lead contact

Requests related to raw experimental data, code, and all academic inquiries can be addressed to the lead contact and corresponding author, Prof. Qingli Li (qlli@cs.ecnu.edu.cn).

### Materials availability

This study did not generate new unique reagents.

### Data and code availability

• The modeling data are available at Zenodo under DOI: https://doi.org/10.5281/zenodo.20339938. Detailed raw data can be requested from X.Y.
• The Python code used for exhaustive feature search, machine learning modeling, and spatial inversion is available at Zenodo under DOI: https://doi.org/10.5281/zenodo.20340056.
• Any additional information required to reanalyze the data reported in this paper is available from the lead contact upon request.

## Acknowledgments

This work is supported by the 10.13039/501100001809National Natural Science Foundation of China (grant nos. 62475072 and 42527804), 10.13039/501100012226Fundamental Research Funds for the Central Universities, and the 10.13039/501100003399Science and Technology Commission of Shanghai Municipality (grant nos. 25xtcx00600 and 22DZ2229004).

## Author contributions

X.Y., conceptualization, methodology, software, formal analysis, writing – original draft, and writing – review and editing; J.W., investigation, data curation, and writing – review and editing; L.S., data curation, validation, and writing – review and editing; S.Z., methodology and visualization; K.D., software and validation; Z.H., resources; Q.L., conceptualization, supervision, project administration, funding acquisition, and writing – review and editing.

## Declaration of interests

The authors declare no competing interests.

## STAR★Methods

### Key resources table

**Table:** 

REAGENT or RESOURCE	SOURCE	IDENTIFIER
**Deposited data**		

Modeling data (COD, NH_3_-N, Chl-a)	This study	https://doi.org/10.5281/zenodo.20339938
Python code for analysis	This study	https://doi.org/10.5281/zenodo.20340056

**Software and algorithms**		

Python 3.9	Python Software Foundation	https://www.python.org/
Scikit-learn	Scikit-learn developers	https://scikit-learn.org/
Rasterio	Mapbox	https://rasterio.readthedocs.io/
XGBoost	DMLC	https://xgboost.readthedocs.io/
CatBoost	Yandex	https://catboost.ai/
LightGBM	Microsoft	https://lightgbm.readthedocs.io/
Matplotlib	Matplotlib Development Team	https://matplotlib.org/

### Experimental model and study participant details

Omitted as our study does not involve biological models.

### Method details

#### Data collection

On October 8, 2024, from 10:00 to 16:00, aerial surveys were co snducted over the experimental area using the Changguang Yuchen AQ600 Pro multispectral camera. Imagery was acquired in five spectral bands: blue (450 ± 15 nm), green (555 ± 13.5 nm), red (660 ± 11 nm), red-edge (720 ± 5 nm), and near-infrared (840 ± 15 nm). The raw digital numbers (DNs) were converted to radiance using laboratory calibration coefficients (Table S1). Subsequently, band-specific linear regression models were established between radiance and surface reflectance using standard reference panels deployed during the flight, allowing retrieval of surface directional reflectance. Geometric and orthorectification corrections were then performed based on high-precision POS data and ground control points. The resulting orthorectified reflectance images were used for subsequent water quality inversion (Figure 1).

The UAV imagery processing was conducted entirely using the Pix4Dmapper software platform, encompassing key steps such as image alignment, dense aerial triangulation, radiometric calibration, orthomosaic generation, and reflectance extraction. The input data included the raw multispectral images across all five bands, high-precision GNSS/IMU positioning and attitude data, ground control point coordinates, and images of simultaneously deployed grayscale reflectance reference panels.

Through synchronized airborne and ground-based sampling, river water samples for COD and NH_3_-N were collected, with 30 ground sampling points systematically established (Figure 1). Water samples were collected at a depth of 20 cm below the water surface using a 30 cm diameter water sampler to ensure the acquisition of representative samples unaffected by surface disturbances. For each sampling point, a circular buffer with a 35 cm radius was created around the central location. Samples were randomly collected five times at different positions within this buffer, and the collected water was transferred to a mixing container and thoroughly blended to ensure homogeneity. Ultimately, 3 L of mixed water sample was obtained from each point for subsequent water quality analysis. After collection, the samples were immediately sealed in opaque brown glass bottles, labeled, and transported in an insulated box to maintain water quality stability. The sampling coordinates were recorded using high-precision GPS. The determination of water quality parameters, COD and NH_3_-N, strictly adhered to the GB3838-2002 standard. The statistical analysis results for various parameters are provided in Table S2.

The UAV imagery has a spatial resolution of 10 cm. Considering the sub-meter local homogeneity of river water quality, we aligned the remote sensing observations with the field sampling scale using a synergistic pixel-and-buffer strategy. A circular buffer with a 35 cm radius was delineated around each RTK-GNSS-located sampling point. Within this buffer, water samples were collected from five random positions using a 30 cm-diameter sampler and thoroughly mixed to form a composite sample representing the bulk water quality of this 70 cm-diameter physical area.

During image processing, a buffer of the identical spatial scale (35 cm radius, covering approximately 38–40 pixels) was applied to each sampling point in the UAV imagery. Because the single laboratory-measured concentration reflects the physically mixed water within the entire buffer, we directly extracted the original reflectance values of the central pixel and 12 randomly distributed pixels within this area (Figure S1). The spectral data of these 13 independent pixels from each site were directly matched with the same corresponding *in situ* water quality parameters (COD and NH_3_-N). This approach respects the spatial representativeness of the ground truth while leveraging the micro-scale spectral variations captured by the ultra-high-resolution imagery. By avoiding mean or median smoothing of pixel reflectance, this strategy effectively expands the sample size without introducing extraneous assumptions, yielding a final dataset of 390 valid spectral-water quality paired samples (30 sampling sites × 13 pixels) for modeling. Importantly, no spatial aggregation (e.g., mean or median) was applied to the reflectance values of these pixels; each retains its original spectral signature while sharing the same water quality label derived from the physically mixed sample. This approach not only effectively mitigates the impact of geolocation errors but also fully leverages the spatial information from UAV imagery, thereby significantly enhancing the representativeness and utilization efficiency of point-based field measurements.^62^

#### Correlation analysis

To improve the accuracy of water quality parameter retrieval, this study employed a systematic spectral band combination analysis approach. Following the band operation rules specified in Table S3, exhaustive combinatorial operations were performed on five original spectral bands. The Pearson correlation coefficient between each resulting band combination and two water quality indicators (COD and NH_3_-N) was then calculated. The correlation coefficient *r* is defined by Equation 1:

(Equation 1) $$r=\frac{\sum \left(x_{i}-\bar{x}\right)\left(y_{i}-\bar{y}\right)}{\sqrt{\sum {\left(x_{i}-\bar{x}\right)}^{2}}\sqrt{\sum {\left(y_{i}-\bar{y}\right)}^{2}}}$$

Where *x*_*i*_ represents the value of the spectral feature combination for the *i* -th sample, and *y*_*i*_ denotes the corresponding measured water quality concentration. $\bar{x}$ and $\bar{y}$ are the mean values of *x*_*i*_ and *y*_*i*_, respectively.

#### Construction of water quality inversion model

Accurate inversion of water quality parameters was achieved in this study by systematically employing five machine learning algorithms, including CatBoost, XgBoost, LightGBM, RF, KNN, and MBLERM. MBLERM employs an advanced Stacking ensemble strategy, integrating four base learners with complementary modeling capabilities: XgBoost, LightGBM, RF, and KNN, and utilizes CatBoost as the meta-learner for intelligent re-learning and optimal weighted fusion of the base model predictions. This architecture leverages the strengths of gradient boosting tree models in handling non-linear, high-dimensional feature interactions (XgBoost/LightGBM), the robust resistance of Random Forest to noise and overfitting, and the sensitivity of KNN to local data structures. Meanwhile, CatBoost effectively suppresses redundant bias during the meta-learning phase through its Ordered Boosting mechanism and adaptive handling of heterogeneous features, significantly enhancing the overall generalization performance.

To fully explore the potential of each model, Bayesian optimization was employed to tune the hyperparameters of CatBoost, XgBoost, LightGBM, Random Forest (RF), and KNN. For the MBLERM model, the hyperparameters of its base learners were also determined via Bayesian optimization.

During the modeling process, for each water quality parameter, the ten spectral features exhibiting the highest correlation with its concentration were first selected. Subsequently, all possible combinations containing three to nine features were exhaustively generated, resulting in a total of 967 distinct input feature sets ($\sum_{k=3}^{9}\left(\begin{array}{c} 10\\ k \end{array}\right)=967$). Each algorithm was trained and optimized across all feature combinations to construct a large-scale model ensemble. Consequently, for each water quality parameter, a total of 6 algorithms × 967 feature combinations yielded 5,802 optimized models, providing a robust dataset for systematically investigating the intrinsic relationship between feature selection and algorithm performance.

The exhaustive feature combination and large-scale model construction strategy enabled a systematic exploration of the feature space across different algorithms. This approach not only comprehensively characterizes the modeling behavior of each algorithm under diverse feature configurations but also provides a solid foundation, within a unified experimental framework, for quantitatively assessing the adaptability, robustness, and generalization ability of various machine learning models in complex aquatic environments. By systematically comparing 5,802 optimized models, the dependence of algorithm performance on input feature combinations was effectively revealed, thereby offering both theoretical guidance and practical pathways for the synergistic design and optimal matching of algorithms and features in high-accuracy water quality parameter retrieval.

#### Accuracy assessment

During model training and validation, the 390 valid samples were partitioned into a training set (312 samples) and a test set (78 samples) at a 4:1 ratio. To ensure spatial representativeness and reliable generalization assessment, the test set was meticulously structured. Specifically, 52 samples were derived from four geographically isolated sampling sites, with all 13 pixels per site (the central pixel and 12 random neighboring pixels) retained entirely within the test set. The remaining 26 test samples were randomly drawn from other independent sampling sites. Crucially, all 30 original sampling sites were treated as indivisible units during data allocation. All 13 pixels corresponding to any single 70 cm-diameter physical water area were assigned exclusively to either the training set or the test set; no single sampling site was split across both subsets. This strict site-level partitioning strategy guarantees physical spatial independence between the training and test data. It effectively mitigates spatial autocorrelation and data leakage arising from local spectral similarity, thereby preventing the overestimation of model performance and enhancing the robustness and extrapolative capability of the evaluation results.

To comprehensively evaluate the prediction performance of all candidate models in the model cluster on the test set, six statistical metrics are selected as accuracy evaluation criteria: Root Mean Squared Error (RMSE), Mean Absolute Error (MAE), Mean Squared Error (MSE), Mean Absolute Percentage Error (MAPE), Coefficient of Determination (R^2^), and Bias, as shown in Equation 2. These metrics collectively assess the overall prediction accuracy and reliability of the model from multiple perspectives.

(Equation 2) $$\begin{array}{l} RMSE=\sqrt{\frac{1}{n}\sum_{i=1}^{n}{\left(y_{i}-{\hat{y}}_{i}\right)}^{2},}\\ MAE=\frac{1}{n}\sum_{i=1}^{n}\left|y_{i}-{\hat{y}}_{i}\right|,\\ MSE=\frac{1}{n}\sum_{i=1}^{n}{\left(y_{i}-{\hat{y}}_{i}\right)}^{2}\text{,} \end{array}\;\begin{array}{l} MAPE=\frac{100\%}{n}\sum_{i=1}^{n}\left|\frac{y_{i}-{\hat{y}}_{i}}{y_{i}}\right|\\ R^{2}=1-\frac{\sum_{i=1}^{n}{\left(y_{i}-{\hat{y}}_{i}\right)}^{2}}{\sum_{i=1}^{n}{\left(y_{i}-\bar{y}\right)}^{2}}\\ Bias=\frac{1}{n}\sum_{i=1}^{n}\left({\hat{y}}_{i}-y_{i}\right) \end{array}$$

Where n represents the number of samples, *y*_*i*_ denotes the observed value of the water quality parameter, $\hat{y}$_*i*_ refers to the predicted value of the water quality parameter, $\bar{y}$_*i*_ indicates the average of the observed values, and $\bar{y}$ represents the average of the predicted values.

#### Feature selection analysis indicators

In machine learning–based water quality parameter inversion, feature selection serves as a critical preprocessing step for enhancing model accuracy and robustness. Traditional approaches typically rely on the strength of correlation between spectral bands and the target parameter, assuming that features with higher correlation coefficients possess stronger predictive capability. However, correlation coefficients reflect only linear relationships, whereas machine learning models—particularly ensemble tree algorithms—are adept at capturing complex nonlinear patterns. Consequently, whether selecting features solely based on the highest correlation coefficients yields optimal performance remains an open scientific question worthy of further investigation. To overcome the limitations of traditional linear correlation–based selection, this study introduces four feature selection indicator factors: the mean correlation coefficient ($\bar{M}$_*PC*_), mean Euclidean distance ($\bar{M}$_*ED*_), mean cosine similarity ($\bar{M}$_*CS*_), and mean spectral angle ($\bar{M}$_*SA*_). These factors quantitatively evaluate the influence of feature selection on model performance metrics—RMSE, MAE, MSE, MAPE, R^2^, and Bias—from multiple perspectives, including linear correlation, distance measurement, directional similarity, and spectral shape variation.

$\bar{M}$_*PC*_ is used to quantify the overall linear correlation strength between a feature combination and the water quality parameter. For any combination of *k* bands, the Pearson correlation coefficients (*r*1,*r*2,…,*rk*) between each feature in the combination and the true water quality parameter measurements are calculated. The absolute values of these correlation coefficients are then taken, and their arithmetic mean is computed, as shown in Equation 3. For example, if a combination includes features *X*_2_,*X*_5_,*X*_6_,*X*_9_, the correlation coefficients *r*_2_,*r*5,*r*6,*r*9 are computed between *X*_2_,*X*_5_,*X*_6_,*X*_9_ and the true water quality sampling values. The arithmetic mean of the absolute values of these four correlation coefficients (*r*_2_,*r*5,*r*6,*r*9) is then calculated.

(Equation 3) $${\bar{M}}_{PC}=\frac{1}{k}\sum_{j=1}^{k}\left|r_{j}\right|$$

Where *k* represents the number of bands in the combination, and *r*_*j*_ denotes the Pearson correlation coefficient between the *j*-th band and the water quality parameter. The calculation method for *r*_*j*_ is given in Equation 4.

(Equation 4) $$r_{j}=\frac{\sum_{i=1}^{n}\left(x_{ij}-{\bar{x}}_{j}\right)\left(y_{i}-\bar{y}\right)}{\sqrt{\sum_{i=1}^{n}{\left(x_{ij}-{\bar{x}}_{j}\right)}^{2}\sum_{i=1}^{n}{\left(y_{i}-\bar{y}\right)}^{2}}}$$

Where *x*_*ij*_ represents the spectral value of the *i*-th sample in the *j*-th band, $\bar{x}$_*j*_ denotes the average value of all samples in the *j* -th band, and *n* is the total number of samples.

The $\bar{M}$_*ED*_ is used to quantify the overall distribution dispersion of sample points in the spectral space formed by a combination of certain bands, as shown in Equation 5.

(Equation 5) $${\bar{M}}_{ED}=\frac{1}{k}\sum_{j=1}^{k}ED_{j}$$

Where *ED*_*j*_ represents the Euclidean distance computed in the *j*-th band, with the calculation method outlined in Equation 6. A larger value of *ED*_*j*_ indicates a more dispersed distribution of the samples in the spectral space of the band combination.

(Equation 6) $$ED_{j}=\sqrt{\frac{1}{n}\sum_{i=1}^{n}{\left(x_{ij}-y_{i}\right)}^{2}}$$

$\bar{M}$_*CS*_ is used to quantify the overall directional similarity between the result sequence of a band combination and the true water quality parameter value sequence. Its value reflects the co-variation trend of the two in the multidimensional space, with the calculation method provided in Equation 7:

(Equation 7) $${\bar{M}}_{CS}=\frac{1}{k}\sum_{j=1}^{k}CS_{j}$$

Where *CS*_*j*_ represents the cosine similarity between the *j* -th band and the water quality parameter, and the calculation method for *CS*_*j*_ is provided in Equation 8. The closer the value is to 1, the more consistent the direction of the spectral curves.

(Equation 8) $$CS_{j}=\frac{\sum_{i=1}^{n}\left(x_{ij}\cdot y_{i}\right)}{\sqrt{\sum_{i=1}^{n}x_{ij}^{2}}\cdot \sqrt{\sum_{i=1}^{n}y_{i}^{2}}}$$

$\bar{M}$_*SA*_ quantifies the similarity in the shape of spectral curves by calculating the generalized angle between spectral vectors. This index is used to assess the overall similarity between the spectral curve shape of a feature combination and the reference curve shape, focusing on capturing the essential features of the data structure, as shown in Equation 9:

(Equation 9) $${\bar{M}}_{SA}=\frac{1}{k}\sum_{j=1}^{k}SA_{j}$$

Where *SA*_*j*_ represents the spectral angle of the *j*-th band, as shown in Equation 10. The smaller the value (typically measured in radians), the more similar the spectral shapes are.

(Equation 10) $$SA_{j}=\arccos\left(\frac{\sum_{i=1}^{n}\left(x_{ij}\cdot y_{i}\right)}{\sqrt{\sum_{i=1}^{n}x_{ij}^{2}}\cdot \sqrt{\sum_{i=1}^{n}y_{i}^{2}}}\right)$$

This section provides a quantitative analysis foundation for systematically analyzing the relationship between feature combination characteristics and model performance by constructing a multidimensional feature selection indicator system.

### Quantification and statistical analysis

All data processing, modeling, and visualization were performed using Python 3.9. Rasterio was utilized for remote sensing image processing. Machine learning models were implemented using Scikit-learn , XGBoost , CatBoost , and LightGBM . Data visualization was conducted using Matplotlib, Seaborn, and Plotly. Statistical significance was set at *p* < 0.05.
