# Supplementary material for: Machine learning water quality inversion via optimal band selection from highly correlated subsets
Source: iScience. 2026 Aug 3;29(8):117027. doi: 10.1016/j.isci.2026.117027 (PMC13470319; doi:10.1016/j.isci.2026.117027)
Supplement: Supplementary file 1 [file mmc1.pdf]

iScience, Volume *29*

Supplemental information

Machine learning water quality inversion via optimal band selection from highly correlated subsets

Xiaonan Yang, Jiansheng Wang, Li Sun, Songjia Zhang, Kun Ding, Zhiping He, and Qingli Li

1 SUPPLEMENTAL INFORMATION

Supplemental Figures

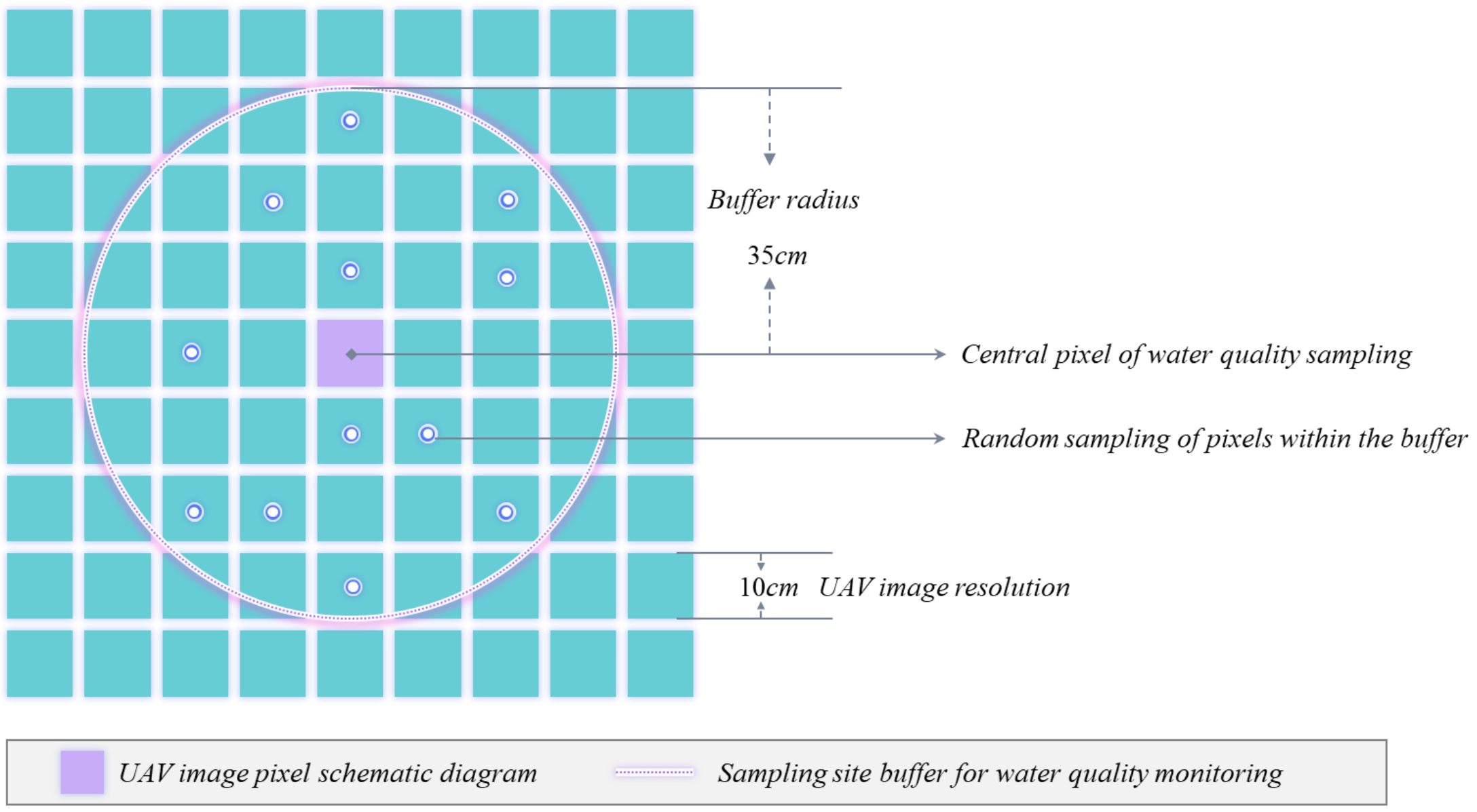

Figure S1. Schematic diagram of random pixel extraction within UAV image buffer zone, Related to Figure 1.

Supplemental Tables

Table S1. Basic Information Table for UAV Flight , Related to Figure 1.

Basic information on multispectral data			
**Collection Time**	2024-10-8 10:00-16:00	**Spatial Resolution**	10cm
**UAV Flight Height**	110m	**UAV Flight Frequency**	11
**On-site Wind Force**	2-3	**Weather Conditions**	Clear and cloudless weather

Table S2. Basic Information on the Five Water Quality Parameters , Related to Figure 1.

Name	Max	Min	Mean	SD
**COD**	15	9	11.87	1.53
**NH_3-N**	5.84	0.218	0.74	1.12

Table S3. Band operation rules table , Related to Table 1.

Type	Band Combination Formulas		
Dual-	$B1 + B2$	$(B1+ B2) / B2$	$e^{(B1+ B2)*B1}$

Band Combinations	*B1 - B2*	*B1 / (B1- B2)*	$e^{(B1+ B2)*B2}$
	*B1 * B2*	*B2 / (B1- B2)*	$e^{(B1- B2) / B1}$
	B1 / B2	*B1 / (B1+ B2)*	$e^{(B1- B2) / B2}$
	(B1- B2) / (B1+B2)	*B2 / (B1+ B2)*	$e^{(B1+ B2) / B1}$
	$((B1)^2- (B2)^2) / ((B1)^2+(B2)^2)$	$e^{B1 + B2}$	$e^{(B1+ B2) / B2}$
	*(B1- B2)*B1*	$e^{B1 - B2}$	$e^{B1 / (B1- B2)}$
	*(B1- B2)*B2*	$e^{B1 * B2}$	$e^{B2 / (B1- B2)}$
	*(B1+ B2)*B1*	$e^{B1 / B2}$	$e^{B1 / (B1+ B2)}$
	*(B1+ B2)*B2*	$e^{(B1- B2) / (B1+B2)}$	$e^{B2 / (B1+ B2)}$
	(B1- B2) / B1	$e^{((B1)2- (B2)2) / ((B1)2+(B2)2)}$	$Log_{10}(1 / B1)$
	(B1- B2) / B2	$e^{(B1- B2)*B1}$	$Log_{10}(1 / B1) - Log_{10}(1 / B2)$
	(B1+ B2) / B1	$e^{(B1- B2)*B2}$	$Log_{10}(1 / B1) + Log_{10}(1 / B2)$
Three-band combination	*B1 + B2 + B3*	*(B1 - B2) * B3*	$e^{B1 * B2 * B3}$
	B1 + B2 - B3	*B1 / (B2+ B3)*	$e^{(B1 - B2) / B3}$
	(B1 - B2 + B3)/(B1 + B2 + B3)	*B1 / (B2- B3)*	$e^{(B1 + B2) * B3}$
	(B1 - B2 + B3)/(B1 + B2 - B3)	$e^{B1 + B2 + B3}$	$e^{(B1 - B2) * B3}$
	*(B1 * B2) / B3*	$e^{B1 + B2 - B3}$	$e^{B1 / (B2+ B3)}$
	(B1 + B2) / B3	$e^{(B1 - B2 + B3)/(B1 + B2 + B3)}$	$e^{B1 / (B2- B3)}$
	*B1 * B2 * B3*	$e^{(B1 - B2 + B3)/(B1 + B2 - B3)}$	$Log_{10}(1 / B1) + Log_{10}(1 / B2) + Log_{10}(1 / B3)$
	(B1 - B2) / B3	$e^{(B1 * B2) / B3}$	$Log_{10}(1 / B1) - Log_{10}(1 / B2) - Log_{10}(1 / B3)$
	*(B1 + B2) * B3*	$e^{(B1 + B2) / B3}$	$Log_{10}(1 / B1) - Log_{10}(1 / B2) + Log_{10}(1 / B3)$
